# A presumed mouse parvovirus with overlooked high toxicity for human primitive CD34^+^ hematopoietic precursors *in vitro* and in bone marrow-humanized mice

**DOI:** 10.1128/spectrum.02339-25

**Published:** 2025-10-27

**Authors:** José C. Segovia, Juan C. Ramírez, Paula Río, Cecilia M. Lotufo, Alberto López-Bueno, Tania Calvo-López, María L. Lamana, Virginia Sandonís, Antonio Balas, José M. Bautista, Paloma Abad, Isabel G. Azcárate, José L. Lopez Lorenzo, Farid A. Jalilian, Razieh Amini, Paula M. Kinnunen, Pikka Jokelainen, Ushanandini Mohanraj, Maria Söderlund-Venermo, José M. Almendral

**Affiliations:** 1 Biomedical Innovation Unit, Centro de Investigaciones Energéticas Medioambientales y Tecnológicas and Centro de Investigación Biomédica en Red de Enfermedades Raras (CIEMAT/CIBERER) 54457, Madrid, Spain; 2 Advanced Therapies Unit, Fundación Instituto de Investigación Sanitaria - Fundación Jiménez Díaz (FIIS-FJD, UAM) 218187 , Madrid, Spain; 3 Departamento de Biología Molecular, Centro Biología Molecular Severo Ochoa (CSIC-UAM), Universidad Autónoma de Madrid 16722, Cantoblanco, Madrid, Spain; 4 Centro de Transfusión de Madrid (CM), Madrid, Spain; 5 Department of Biochemistry and Molecular Biology, Research Institute Hospital 12 de Octubre (Imas12), Universidad Complutense de Madrid 16734, Madrid, Spain; 6 Faculty of Health Sciences, Rey Juan Carlos University 619352, Alcorcón, Spain; 7 Hospital Fundación Jiménez Díaz 16436 , Madrid, Spain; 8 Department of Virology, Faculty of Medicine, Hamadan University of Medical Sciences 48430, Hamadan, Iran; 9 Research Center for Molecular Medicine, Hamadan University of Medical Sciences 48430, Hamadan, Iran; 10 Faculty of Veterinary Medicine, University of Helsinki 3835, Helsinki, Finland; 11 Infectious Disease Preparedness and One Health, Statens Serum Institut 4326, Copenhagen, Denmark; 12 Department of Virology, University of Helsinki 3835, Helsinki, Finland; University of Manitoba, Winnipeg, Manitoba, Canada

**Keywords:** human parvovirus, minute virus of mice, CD34+ targeting, human hematopoietic toxicity, virus host range, parvovirus evolution, capsid tropism determination, serology, epidemiology

## Abstract

Two strains of the minute virus of mice (MVM) parvovirus (of the species *Protoparvovirus rodent1*), named i (immunosuppressive) and p (prototype), show disparate tropism and pathogenicity in mice. Unlike the virulent MVMi, the MVMp is attenuated and infection-restricted in primary mouse hematopoietic cells; however, it does infect human transformed, established, and stem cells. We thus seek here a putative MVMp natural tropism to humans compared to MVMi. Infectious purified MVMp virions suppress *in vitro* the clonogenic potential of human committed (erythroid and myeloid) and primitive CD34^+^ progenitors, regardless of the cell source (umbilical cord blood, G-CSF mobilized peripheral blood, or bone marrow), in a multiplicity of infection-dependent manner. Furthermore, primitive CD34^+^ progenitors in culture are permissive to the MVMp cytotoxic NS-1 protein expression and the synthesis of viral genome replicative intermediates. None of these features is shared by MVMi. In consistency, MVMp suppresses the pool of CD34+ progenitors in bone-marrow-humanized immunodeficient NSG mice and severely compromises their survival, whereas in basal NSG mice, it replicates to several thousand-fold lower levels than MVMi and undergoes evolution to variants carrying genetic changes at the capsid tropism determinant binding sialic acid types. We suggest that MVMp may have originated from a MVMi-like mouse parvovirus upon adaptation to human sialic acid receptors in immunocompromised individuals. Extensive serological and genetic screens failed to demonstrate circulating MVMp in Eurasian and African human populations, including hematopoietic patients. Nevertheless, the high MVMp cytotoxicity to human primitive hematopoietic progenitors and rapid evolvability deserve further epidemiological studies.

**IMPORTANCE:**

Assigning the genuine natural viral host range may be uncertain without testing the susceptibility of primary cells. *Parvoviridae,* a family of ssDNA icosahedral viruses, harbors several members infecting humans, but only parvovirus B19 of the *Erythroparvovirus* genus shows tropism for human hematopoietic progenitors. Here, we evaluate the tropism of the MVMi and MVMp parvoviruses for humans, which are assumed to be mouse pathogens. We show that MVMp is remarkably cytotoxic for human primitive CD34+ and committed erythroid and myeloid hematopoietic progenitors. Infection of basal and bone marrow-humanized immunodeficient mice shows the emergence of viral strain-specific genetic changes at the capsid domain binding sialic-acid receptors, denoting adaptation to the respective host hematopoiesis. Although a large epidemiological survey failed to identify circulating MVMp sequences or antibodies in human populations, its high toxicity to human hematopoietic progenitors of different lineages and primitiveness and rapid evolutionary capacity demand in-depth characterization of its potential pathogenicity for humans.

## INTRODUCTION

Assignment of the genuine host range of virus isolates and metagenomic sequences is complicated by the limited availability of primary cells and the commonly promiscuous permissiveness to heterologous viruses of established immortalized and transformed cell lines. The *Parvoviridae* is a family of small single-stranded DNA viruses (1) infecting a broad range of animal species in nature (2–5). These viruses require factors expressed during the S-phase of the cell cycle for their multiplication (6–9). Correspondingly, the extent of parvovirus infection and pathogenicity is linked to the proliferative state of the cells in tissues and the developmental state of the host (10), making the hematopoietic system a common parvovirus target (11, 12).

In humans, several members of the *Parvoviridae* belonging to distinct genera may infect with diverse prevalence and are associated with a spectrum of diseases that still needs to be established (13–16). Parvovirus B19 (B19V) of the genus *Erythroparvovirus* is so far the only parvovirus associated with severe human hematopoietic dysfunctions (14, 17). In children, B19V is the etiological agent of the “erythema infectiosum” or fifth viral disease, potentially causing several congenital abnormalities and hydrops fetalis (18). In adults, patients suffering from hemoglobinopathies or immunosuppression are especially susceptible to B19V infection, causing red cell aplasia and aplastic anemia (19, 20). The B19V circulates worldwide; it replicates in the bone marrow (BM), resulting in extremely elevated levels of viremia and systemic infection associated with multiple clinical manifestations (21), and its genome or pieces can persist lifelong in several human tissues as bone marrow and tonsillar B cells (22–27). *In vitro*, B19V suppresses the colony-forming capacity of the human erythropoietic precursors (erythroid colony-forming unit [CFU-E] and erythroid burst-forming unit [BFU-E]) (28) but not that of the myeloid precursors (granulo-macrophage colony-forming unit [CFU-GM]), and primitive progenitors fail to support virus replication (29). The virus shows marked tropism for progenitors of the erythroid lineage, the susceptibility of which increases with precise differentiation stages (30). These requirements may partly explain the successful though restricted multiplication of B19 in human bone marrow cultures (31, 32), despite the high levels of viremia frequently observed in natural infections (21).

In mice, two strains of minute virus of mice (MVM), belonging to the recently re-named *Protoparvovirus rodent1* species (1), have served as major classical molecular models for the *Parvoviridae*, the prototype (p) and the immunosuppressive (i), which show distinct tropism and pathogenicity in mice despite their high genetic similarity (33, 34). MVMi was isolated from a culture of the mouse EL-4 lymphoma (35). It can inhibit T cell-mediated functions (36) and cytotoxically infect committed CFU-GM, BFU-E, megakaryocyte progenitors (CFU-MK), and primitive (CFU-S) mouse clonogenic hematopoietic precursors (37, 38), as well as long-term repopulating mouse hematopoietic stem cells (39). In newborn mice, MVMi impairs brain developing areas (40), mediates a mild reduction of hematopoietic committed precursors (41), as well as an involution of hepatic erythropoietic foci (42). In adult severe immunodeficient mice (scid), MVMi causes a persistent bone marrow infection, leading to dysregulated erythropoiesis and lethal leukopenia (43), which, when subjected to passive antibody-mediated therapies, unraveled a powerful virus evolution capacity (44, 45). These reports clearly assign MVMi as a virulent mouse pathogen. Other members of this group of viruses, which have become more prevalent in animal facilities recently, are the MVMc and MVMm strains (46), the latter being the only strain isolated directly from mice and exhibiting a hematopoietic pathogenicity similar to that of MVMi (47).

However, the prototype strain of the MVM (MVMp) was isolated from mouse adenovirus-infected transformed cells (48), but never from a wild mouse or a rodent laboratory facility. MVMp has been traditionally considered a mouse virus because it efficiently infects the established A9 mouse fibroblast cell line (49), but this cell line harbors the p53-V173L mutation crucial for permissive infection (50), thereby questioning the presumed MVMp natural tropism for mouse fibroblasts. Indeed, MVMp gene expression is severely restricted in primary mouse embryo fibroblasts (51), which required multiple passages in coinfections with mouse adenovirus and polyomavirus (48, 52), two oncogenic viruses able to provide helper functions to other parvoviruses (53). Moreover, in mice, MVMp is attenuated in the newborn (54), unable to infect primary mouse hematopoietic precursors (37), and its pathogenicity in adult *scid* mice requires evolution to genetic variants carrying MVMi-like mutations (55, 56). Intriguingly, MVMp may efficiently infect many types of human-transformed cell lines (57, 58) and even primary human glioblastoma stem cells (50). These reports collectively question a genuine mouse natural host range for the MVMp strain.

In the present investigation, we undertook a careful examination of a potential MVMp natural tropism to humans, in comparison with MVMi, by exploring its capacity to directly infect primary human hematopoietic progenitors *in vitro,* as well as its pathogenicity and evolutionary trend in bone marrow-humanized immunodeficient mice. The study was accompanied by a large epidemiological survey on the MVMp presence in human populations of different continents, involving serological and genetic screenings of multiple types of hematopoietic and immunocompromised patients. Our findings, supporting MVMp’s potential pathogenicity for humans and an evolutionary origin from a mouse MVMi-like parvovirus, are discussed.

## RESULTS

### Infectious MVMp, but not MVMi, inhibits the colony-forming ability of human hematopoietic progenitors

**Fig 1:**
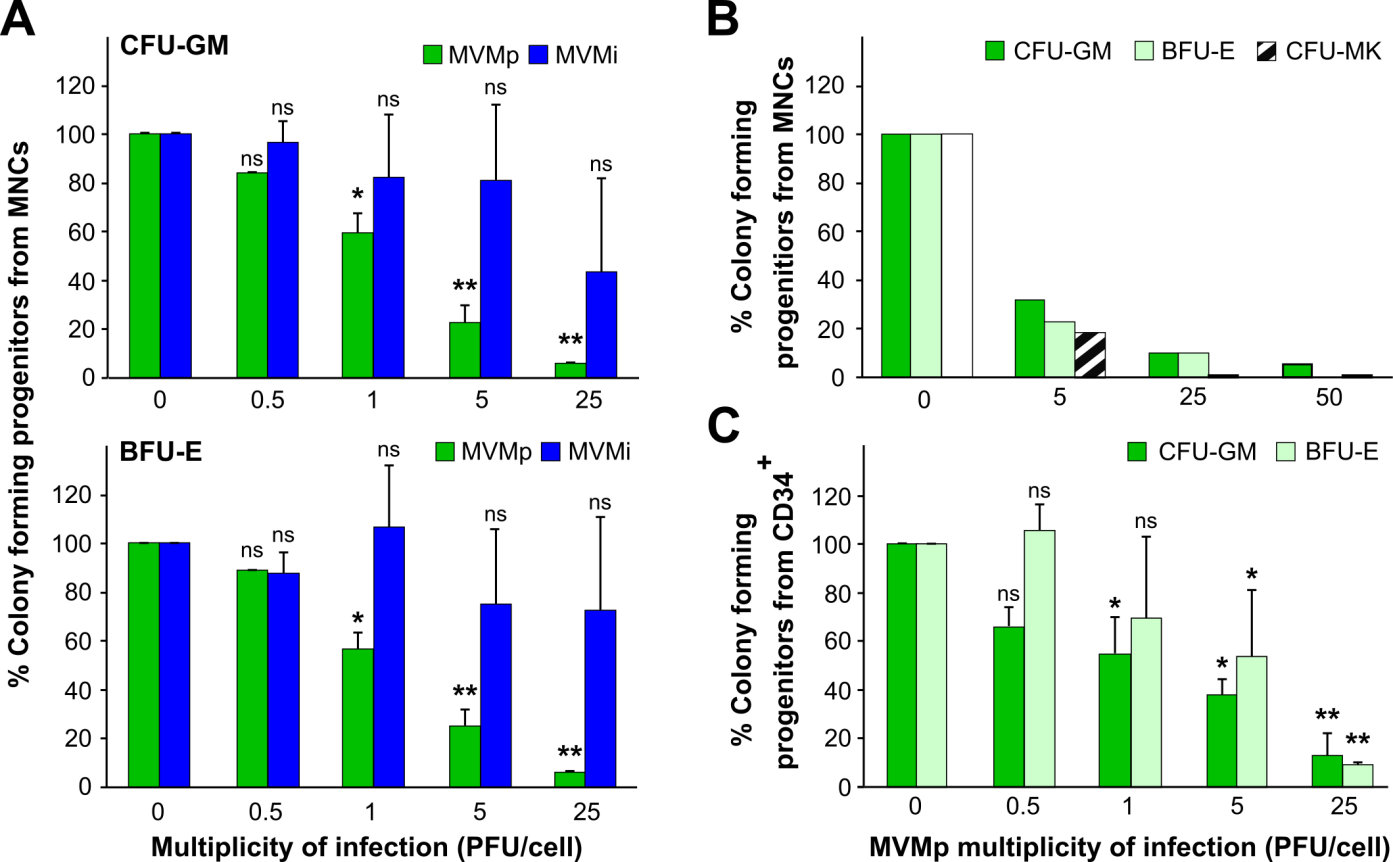
Human hematopoietic progenitors are susceptible to the parvovirus MVMp but not to the MVMi strain. Human MNCs or CD34^+^ progenitors obtained from umbilical cord blood cells were infected at different MOIs with MVMp or MVMi and seeded in semisolid media to allow the growth of CFU-GM, BFU-E, and CFU-MK colonies. (**A**) Susceptibility of myeloid CFU-GM (upper) and erythroid BFU-E (lower) progenitors from human MNCs to MVMp and MVMi at the indicated MOI. (**B**) A representative experiment in which the susceptibility to MVMp of three hematopoietic progenitors from human MNCs committed to three lineages, CFU-GM (filled bars), BFU-E (empty bars), and CFU-MK (striped bars), was evaluated. (**C**) Susceptibility to MVMp of CFU-GM and BFU-E progenitors from CD34^+^ cells. The MOI is indicated below. Colonies were scored after 14 days in culture. Data in panels A and C represent the mean ± SD of three different experiments. Statistical significance in comparison to the mock-infected cells is represented by asterisks (*P < 0.05; **P < 0.01; and ns, not significant).

As a first analysis on the susceptibility of the human hematopoiesis to the MVMi and MVMp strains, mononuclear cells (MNCs) from human cord blood samples were incubated *in vitro* at different multiplicities of infection (MOIs) with purified stocks of these viruses and cultured thereafter in semisolid enriched medium to allow the growth of colony-forming unit-granulocyte-macrophage (Fig. 1A, upper), burst-forming unit-erythrocyte (Fig. 1A, lower) and colony-forming unit-megakaryocyte (Fig. 1B) progenitors. A clear MOI-dependent inhibition was observed when the MNCs were infected with MVMp in the three lineage-committed progenitors. On the contrary, only a minor and non-statistically significant decrease in the colony growth was observed when MVMi was inoculated at identical MOIs, even at the high 25 plaque-forming units (PFUs)/cell dose.

The MNCs constitute a very heterogeneous cell population, including stem cells, committed progenitors, and mature lymphocytes, monocytes, and others. Among them, the multi-lineage clonogenic capacity of this population relies on the more primitive CD34^+^ subpopulation. To exclude the possibility that the observed inhibition of committed progenitors’ clonogenicity could be due to the expression of cytotoxic mediators produced by the infection of non-CD34^+^ cells present in the culture, we studied the effect of direct viral interaction with CD34^+^ purified cells. As observed in Fig. 1C, the clonogenic capacity of CD34+ cells to form CFU-GM and BFU-E was inhibited by MVMp in a similar MOI-dependent manner as that of the MNC population. Of note, minor, yet non-significant differences were observed between the mock- and the MVMi-infected cultures under comparable conditions. These results strongly suggest that the colony growth inhibition was due to a direct interaction of the purified MVMp viral particles with the CD34+ primitive cells. This interaction would inhibit the proliferative activity of the CFU-GM, BFU-E, and CFU-MK committed progenitors.

**Fig 2:**
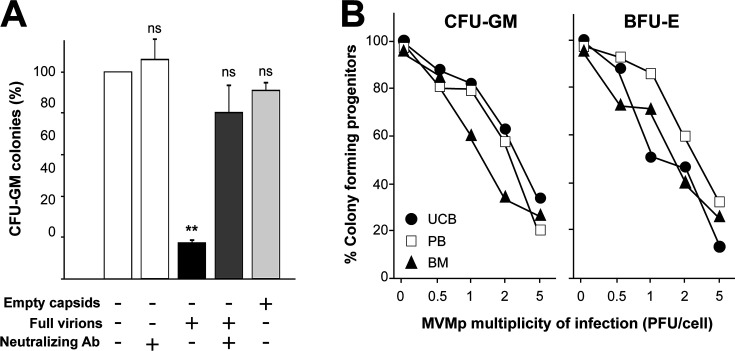
Inhibition of the colony-forming ability of human committed progenitors by MVMp requires infectious virus and is independent of the source of hematopoietic cells. (**A**) Suppression of myeloid progenitor susceptibility to MVMp by previous virus neutralization with antibody. Shown is the colony-forming capacity of CFU-GM progenitors from human MNCs after the different treatments indicated in the footnote of the graphic. Data represent the mean ± S.D. of three independent experiments. (**B**) Susceptibility of the colony-forming capacity of CFU-GM (left panel) and BFU-E (right panel) progenitors in MNCs from different sources (umbilical cord blood [UCB], G-CSF mobilized peripheral blood [MPB], and bone marrow) to the MVMp infection at the indicated MOI, scored at 14 dpi. Statistical significance in respect to the mock-infected culture: ** *P* value < 0.01; ns, not significant.

To further elaborate on the nature of the inhibitory phenomenon, two controls were added to preclude misinterpretations by putative indirect factors: a previous incubation with an anti-MVM neutralizing antiserum (see Materials and Methods) and an inoculation with purified MVMp empty capsids adjusted to equivalent protein content by hemagglutination (see Materials and Methods) compared to the infective virus-containing inoculum. As shown in Fig. 2A, cultures of MNC cells infected by MVMp at MOI 5 yielded close to fivefold lower numbers of CFU-GM colonies compared to mock-infected cultures, whereas none of the controls impaired the CFU-GM colony-forming ability of the hematopoietic progenitors. These results suggest that the inhibition of human progenitors observed with MVMp requires the presence of genuine infectious virus, as this effect cannot be merely mimicked with non-infectious purified empty capsids, nor be caused by a putative toxic effect of the viral structural components.

To enhance the biological significance of this set of experiments, we addressed whether the observed susceptibility of the human primitive precursors to the direct interaction with MVMp was a unique characteristic of the cord blood cells because of their primitiveness, or it may be shared with hematopoietic progenitors from adult sources. For this, human MNCs from three different sources (umbilical cord blood [UCB], bone marrow, or G-CSF-mobilized peripheral blood) were infected with purified MVMp at increasing MOI up to 5 PFU/cell, and the number of the CFU-GM and BFU-E colonies arising was scored in the respective cultures. As depicted in Fig. 2B, no major differences in the inhibition of the colony-forming abilities induced by MVMp were observed between the cell sources. These experiments collectively indicate that infectious MVMp virions inhibit the clonogenic capacity of human CD34+ primitive hematopoietic progenitors in a MOI-dependent manner, regardless of the source of the mononuclear fraction containing CD34^+^ cells used.

### MVMp genome replication and expression in human myeloid progenitors

**Fig 3:**
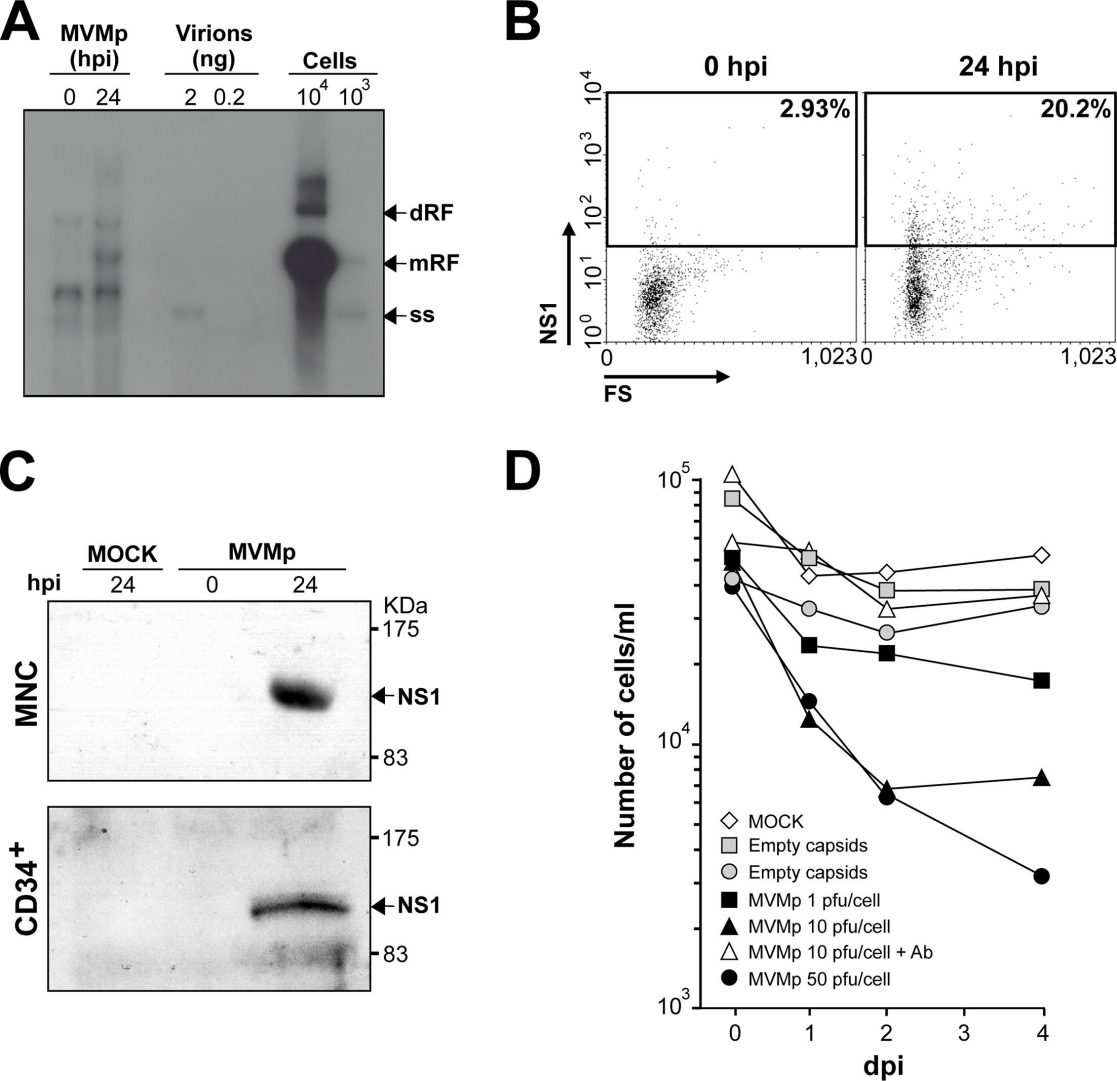
MVMp genome replication and non-structural gene expression in human hematopoietic progenitors. (**A**) MVMp genome replication in human hematopoietic cells. Southern blot analysis of viral genomes in CD34^+^ human progenitor cultures infected by MVMp at an MOI of 5. Samples of 2 × 10^4^ cells were collected at 0 and 24 hpi and analyzed for viral DNA hybridizing with a MVM 32P-DNA probe. Controls: virions, ssDNA genomes extracted from purified virus preparations (an estimation of 2 and 0.2 ng of ssDNA viral genomes were loaded); cells, low-molecular-weight DNA isolated from the indicated number of A9 fibroblasts infected at an MOI of 5 with MVMp. The viral replicative forms (single-stranded viral genomes; monomeric *mRF* and dimeric *dRF* replicative intermediates) are indicated to the right. The filter was exposed to autoradiography with an intensifying screen for 24 hours. (**B**) Parallel analysis for the NS-1 protein expression by flow cytometry in the same MVMp-infected CD34^+^ culture using a specific monoclonal antibody. The positive gate was established by staining with the corresponding antibody isotype. (**C**) Western blot analysis of NS1 expression in the indicated human hematopoietic cells using a rabbit anti-NS-1 polyclonal antibody. (**D**) MVMp inhibits human CD34^+^ viability in culture. The figure shows the number of cells in cultures of human CD34^+^ progenitors inoculated with MVMp at the indicated MOI and conditions. Symbols: (△), MOI 10 in the presence of neutralizing anti-MVMp capsid polyclonal antibody (1/10^3^); inoculations with (●) high (2 µg) and (■) low (2 ng) amounts of purified empty MVMp capsids. Cell viability was measured daily using trypan blue dye exclusion.

To investigate the basis of the inhibition of the colony-forming ability of human hematopoietic progenitors by infectious MVMp, the main macromolecular parameters of the virus life cycle were determined in liquid cultures of these cells. Human primitive CD34^+^ and MNC hematopoietic progenitors obtained from umbilical cord blood cells cultured in the presence of interleukin 3 (IL-3), interleukin 6, and Stem Cell Factor (SCF) to stimulate proliferation were infected at high MOI 5 or mock-infected and sampled for molecular analyses. Southern blot analysis of MVMp genome replication in CD34+ cultures shows a prominent band at 24 hours post-infection (hpi) that was not observed at 0 hpi, corresponding in size to the viral mRF replicative intermediate form (Fig. 3A, left lanes). Although the level of mRF synthesis was substantially lower than that obtained in a similar number of fully permissive A9 mouse fibroblasts (Fig. 3A, right lanes), a patent permissiveness of the CD34+ cells to the synthesis of the MVMp major genome replicative intermediate could be demonstrated. However, no apparent increase in the accumulated single-stranded viral DNA (ss) genomes could be appreciated between the 0 and 24 hpi interval, suggesting a defect of the CD34^+^ cells in packaging the viral DNA replicative forms into capsids.

The transcriptional activity of viral genomes in CD34+ cultures was analyzed through the expression of the multifunctional non-structural NS-1 protein required for viral cytotoxicity and genome replication (2, 59, 60). Flow cytometric analysis demonstrated NS1 expression in close to 20% of the CD34+ cells at 24 hpi (Fig. 3B). In consistency, the expressed NS1 was resolved as a unique protein species of expected size by western blot in CD34+, as well as in MNC cultures at 24 hpi (Fig. 3C).

The effect of the viral gene expression on the viability of human committed and primitive myeloid progenitors over time was next studied by infecting CD34^+^ cells at different MOIs. As shown in Fig. 3D, a marked reduction in the cellularity of the infected culture was observed since 1 day post-infection (dpi) in comparison with mock-infected cultures. This reduction in viability was more severe in infections at a high MOI at 2–4 dpi. However, as observed above in the CFU study, the viability of the cells was virtually unaffected if the virus infectivity was neutralized by an anti-capsid antibody prior to inoculation, or high amounts of empty viral capsids, which compete for the same receptor (61), were inoculated instead (Fig. 3D). These results suggest that the expression of the highly cytotoxic NS1 protein (2, 59, 60) may determine the decay of viability of the human hematopoietic cells. Interestingly, the titer of infectious virus production in these cultures, either considering intracellular or extracellular viral particles at 2–4 dpi, was below detectable levels in several independent determinations (data not shown). These data are in accordance with the failed increase in ssDNA viral genome accumulation at late post-infection times (Fig. 3A), suggesting that MVMp follows a cytotoxic abortive infection cycle in human myeloid cultures.

### MVM interferes with the human primitive hematopoiesis in BM-humanized mice

**Fig 4:**
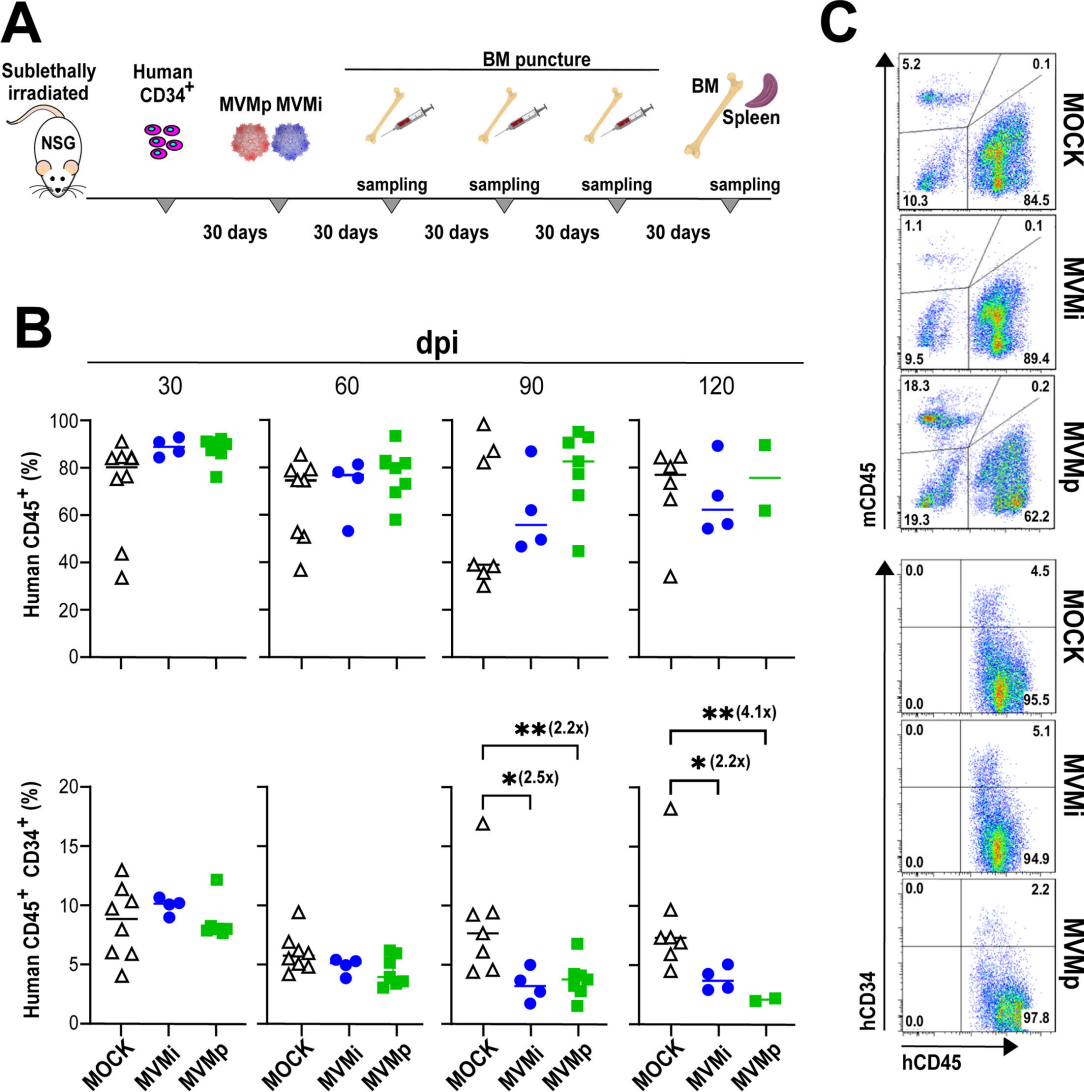
Hematopoietic parameters in MVM-infected BM-humanized immunodeficient mice. (**A**) General scheme of NSG mice handling. The timeline of human CD34+ cell transplantation in sub-lethally irradiated mice, viral strain infection, and sampling of hematopoietic organs is outlined. (**B**) Impact of the MVM strains on human hematopoiesis in BM-humanized mice. Shown are percentages of total CD45+ human hematopoietic cells (upper), or the subpopulation of primitive CD34+ precursors (lower) monitored by flow cytometry in control mock, MVMp-, and MVMi-infected BM-humanized NSG mice. Numbers in parentheses indicate the factor of human precursor decay by MVMi or MVMp infection compared to those in mock mice. *P value < 0.05; **P value < 0.01. (**C**) A representative example of the differentially unbalanced mouse/human hematopoiesis in MVMp- and MVMi-infected mice at 120 dpi. Left: total mouse (mCD45) versus human (hCD45) hematopoietic cells; right: primitive human CD34+ precursors versus mouse total (mCD45) hematopoietic cells. Numbers within the window indicate the percentage of positive cells stained by the respective antibodies.

Attempting to characterize the capacity of the MVM strains to interact with the human hematopoiesis at the organismal level, immunodeficient (NOD.Cg-*Prkdc^scid^Il2rg^tm1Wjl^*/ThomJ [NSG]) mice were transplanted with human cord-blood CD34+ cells to generate a xenogenic human hematopoiesis prior to intravenous infection with purified sterile-filtered MVMp and MVMi virions. Mice were then monitored by BM sampling along 120 dpi for human and mouse hematopoietic progenitors, virus replication and genetics, and survival (see scheme of the experimental procedure outlined in Fig. 4A). The proportion of human CD45+ cells in BM aspirates at 30–120 dpi showed a general high level of engraftment of human cells, although with ample heterogeneity ranging from 40% to 90%, both in control non-infected as well as in MVMi or MVMp infected transplanted mice (Fig. 4B, upper panels). However, significant differences in the pool of primitive human hematopoietic progenitors were observed by flow cytometry quantitation along the infection time. As shown in the lower panels of Fig. 4B, uninfected mice exhibited wide heterogeneity in the percentage of CD34+ progenitors with respect to total CD45+ human cells, ranging from 5% to 18%, whereas the heterogeneity was narrower in infected mice. The proportion of human CD34+ progenitors in mock and infected mice was similarly high, in the range of 10% at 30 dpi, although it declined to close to 5% at 60 dpi. However, as the infection progressed to later time points, while uninfected mice maintained a fairly high proportion of human primitive CD34+ progenitors, this proportion significantly declined to values that were more than twofold lower in MVMp- and MVMi-infected mice at 90 dpi, and further, the MVMp-infected surviving mice significantly showed the lowest CD34+ proportion (close to 2%) at 120 dpi (Fig. 4B, bottom right). As exemplified for the 120 dpi time point, the global response of the bone marrow to MVMi and MVMp infection may drastically vary: whereas the pool of bone marrow mCD45+ cells was depleted by MVMi but enhanced by MVMp infection with respect to that of uninfected mice (Fig. 4C, left), the pool of CD34+ cells in the bone marrow of uninfected and MVMi-infected mice remained at a higher proportion than in the MVMp-infected mice (Fig. 4C, right). In summary, despite the heterogeneity of the data, likely reflecting the complexity of the MVM strain interactions with the chimeric hematopoiesis of the BM-humanized mice, a viral suppressive effect on human primitive hematopoietic precursors could be drawn, which was more severe in MVMp infections.

### Replication, pathogenicity, and evolution of the MVMp and MVMi strains in immunodeficient basal and BM-humanized mice

**Fig 5:**
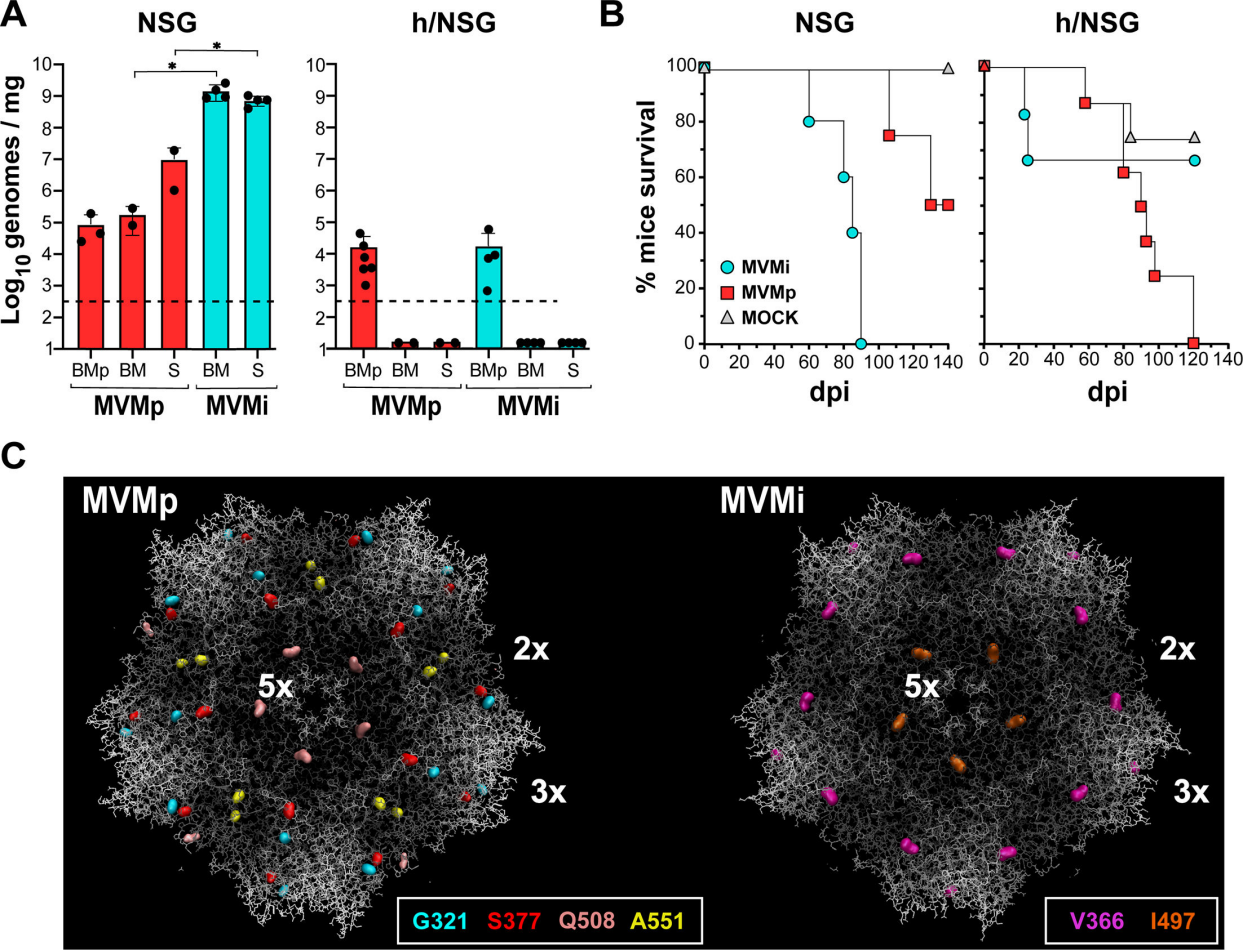
MVM strain replication, pathogenicity, and evolution in normal and BM-humanized immunodeficient mice. (**A**) Quantitation of MVM genomes in mice. Viral genomes quantitated by qPCR with MVM-specific primers in low-molecular DNA isolated from the indicated organs from basal (left) and bone marrow-humanized (right) NGS mice. Shown are data obtained from BMp, BM, and spleen (S) from MVMp- and MVMi-infected mice. Samplings were performed at the post-infection times outlined in Table 1. The dotted line marks the reliable detection limit of the qPCR. Statistical significance: *P* = 0.0332 and *P* = 0.0107, respectively (*P < 0.1). (**B**) Kaplan-Meier representation of NSG and h/NSG mouse survival subjected to the indicated treatments along 150 days post-infection. (**C**) Localization of the amino acid changes in the MVM capsid arising along the evolution of both viral strains in basal and BM-humanized NGS mice. The 3-D crystal structure of the MVMp and MVMi capsids is illustrated using Visual Molecular Dynamics and the PDB coordinates 1Z14 and 1Z1C, respectively. Amino acid changes are highlighted by arbitrary colors indicated for each type of residue.

To dive into the features of the MVM strain interaction with human and mouse hematopoiesis at the organismal level, the extent of viral replication and genetics was determined in basal (NSG) and BM-humanized (h/NSG) main hematopoietic organs (BM and spleen). In NSG mice (Fig. 5A, left), MVMi replicated the genome to very high levels in the hematopoietic organs at 60 dpi, whereas the MVMp did not, since we found more than 2 log less accumulated genomes in the BM and spleen. In consistency, the survival of NSG mice infected with MVMi was drastically reduced from 60 dpi, as previously reported in the infection of immunodeficient *scid* mice (43), with no surviving animals beyond 90 dpi. In contrast, MVMp only compromised survival after 110 dpi, and 50% of the animals still survived at 150 dpi by the end of the experiment (Fig. 5B, left). However, the outcome of the infections in mice transplanted with human CD34+ hematopoietic cells (h/NSG) drastically differs and was, to a large extent, the opposite. Both strains replicate to a similar low level in bone marrow punctures (BMp) sampled at 60 dpi from h/NSG mice (Fig. 5A, right), implying only a fivefold reduction in respect to the replication level obtained in the hematopoietic organs of NSG mice at 90 dpi for MVMp, but as high as five logarithmic units for MVMi. At 120 dpi, though, viral genomes could not be reliably detected in the hematopoietic organs of h/NSG mice, suggesting efficient clearance of both viral strains by a functional immune response developed from the CD34^+^ transplants. Notably, the h/NSG mice survival was hampered by MVMp to a much greater extent than by MVMi (Fig. 5B, right), as the MVMi infection lethality did not significantly diverge from that of uninfected transplanted mice in the long term, but MVMp infection drastically reduced mice survival beyond 100 dpi to background levels.

The genetics of the MVM strains replicating in the hematopoietic organs of NSG and h/NSG mice was analyzed to trace any evidence of selection that could shed light on their natural host range. The study was mainly focused on a region of the VP proteins, configuring a dimple at the twofold axis of the 3-D structure of the MVM strain capsid (62, 63), where the domain binding sialic acid receptors (sbd) localizes (55, 64), and mutations that change MVM tropism *in vitro* (65) and pathogenicity in mice (56) were identified. Consensus Sanger sequences identified the selection of some coding mutations changing VP2 amino acid residues in the virus populations harvested from cohorts of mice (Table 1; Fig. S1), which emerged at variable frequencies denoted by heterogeneities in the sequence profiles (Fig. S1). MVMi replicating to high levels in NSG mice up to 90 dpi did not develop genetic changes within this capsid domain, although the I497M mutation emerged in all samples (Table 1, lower). In sharp contrast, the MVMp populations growing in NSG mice acquired, despite their much lower replication level (Fig. 5A, left), the S377N mutation at high frequency in two mice, and the Q508L and G321E mutations in one other mouse at high and low frequencies, respectively, while a third mouse did not show significant genetic heterogeneity across this region (Table 1, upper). In BM-humanized mice, despite the drastically limited extent of replication of both viral strains (Fig. 5A, right), some reliable genetic changes accumulated to low levels could be detected by nested PCR (Table 1) and confirmed in repeated sequences (Table S1, lower). The MVMi populations show, at high frequency, the V366L change in all the hematopoietic samples of one mouse, as well as the above-mentioned I497M change, albeit at lower frequency than in the no BM-chimeric mice (Table 1*,* bottom). The MVMp populations harvested at different dpi from the BM of all five analyzed mice consistently acquired the A551V change at a high frequency. A transient K536E mutation detected in a BMp of one mouse at 60 dpi did not prevail at later post-infection times. In the capsid 3-D crystal structures of the MVM strains, most mutations arising in NSG mice (V366L in MVMi; G321E, S377N, and A551V in MVMp) localize within the sbd at the capsid twofold axis (Fig. 5C). Interestingly, the Q508L (in MVMp) and the I497M (in MVMi) mutations that are also exposed to the solvent localize very close in the deep groove surrounding the fivefold axis of symmetry.

**TABLE 1:** VP2 amino acid changes in MVM variants recovered from the organs of basal and BM-humanized NSG mice

						321^*d*^	377^*d*^	366^*d*^	497^*d*^	508^*d*^	536^*d*^	551^*d*^
					MVMp	G	S	V	M	Q	K	A
Mouse #	Hem^*a*^	Organ^*b*^	dpi	PCR^*c*^								
1	m	BMp	100	I		E (8)	N (21)	V	M	Q	K	A
2	m	BMp	100	I		G	N (26)	V	M	L (21)	K	A
		BM	140	I		G	N (76)	V	M	L (75)	K	A
		S	140	I		G	N (73)	V	M	L (93)	K	A
3	m	BMp	100	I		G	S	V	M	Q	K	A
		BM	140	I		G	S	V	M	Q	K	A
		S	140	I		G	S	V	M	Q	K	A
8	h	BMp	60	IIA		G	S	V	NA	NA	NA	NA
9	h	BM	120	IIB		G	S	V	M	Q	K	V
10	h	BMp	60	IIB		G	S	V	M	Q	K	V
11	h	BMp	60	IIB		G	S	V	M	Q	E (20)	V
		BM	120	IIB		G	S	V	M	Q	K	V
12	h	BMp	60	IIB		G	S	V	M	Q	K	V
13	h	BMp	60	IIB		G	S	V	M	Q	K	V

					MVMi	E	S	V	I	Q	K	V
4	m	BM	90	I		E	S	V	M	Q	K	V
5	m	BM	90	I		E	S	V	M	Q	K	V
6	m	BM	90	I		E	S	V	M	Q	K	V
		S	90	I		E	S	V	M	Q	K	V
7	m	BM	90	I		E	S	V	M	Q	K	V
		S	90	I		E	S	V	M	Q	K	V
14	h	BMp	60	IIB		E	S	V	I	Q	K	V
		BM	120	IIB		E	S	V	M (17)	Q	K	V
		S	120	IIA		E	S	V	NA	NA	K	NA
15	h	BM	120	IIB		E	S	V	M (52)	Q	K	V
16	h	BMp	60	IIA		E	S	L (57)	NA	NA	K	NA
		BM	120	IIB		E	S	L	M	Q	K	V
		S	120	IIA		E	S	L (90)	NA	NA	K	NA
17	h	BMp	60	IIB		E	S	V	M (80)	Q	K	V
		BM	120	IIA		E	S	V	NA	NA	K	NA
		S	120	IIA		E	S	V	NA	NA	K	NA

^*a*^ Hem: hematopoiesis in normal (m) and BM-humanized (h) NSG mice.

^*b*^ S, spleen.

^*c*^ PCR: genome region subjected to Sanger sequencing: I, 2,722–4,490 nt; IIA (nested PCR), 3,668–4,058 nt; and IIB (nested PCR), 2,793–4,441 nt.

^*d*^ Numbers: VP2 positions of MVM capsid affected by mutations. Heterogeneity at certain positions in the consensus sequence of the viral genome populations is indicated by a number in parentheses. This number corresponds to the percentage of area in the mutation peak in respect to that of the wt sequence estimated by triangulation in the chromatograms using the Fiji software (average of two to three sequences). NA, not applicable, position not sequenced in this sample.

### Epidemiology of MVM in human populations

To look for MVMp that might currently be circulating in human populations, we conducted an epidemiological analysis of serum samples originating from European, Asian, and African countries. The first analysis was carried out by serology, as this technique is generally more sensitive than others to detect the circulation of an infectious virus, as the immune fingerprint is more durable than physical viral presence. Serum samples from 1,032 individuals, including both healthy individuals and patients affected by several hematopoietic or other diseases, were collected for epidemiological IgG screening using two different enzyme immunoassay (EIA) systems (see Materials and Methods). The collection of sera from adults in Finland and Iran has previously been analyzed for IgG of three MVMp-related human protoparvoviruses, bufa-, tusa-, and cutavirus (66, 67). As shown in Table S3, the MVMp IgG EIA showed no cross-reactivity with any of the other three protoparvoviruses. Some serum samples showed IgG absorbances > 0.5 but could not be blocked by purified virus-like particles (VLPs) of MVMp, meaning that they were non-specific reactions, an unclear effect previously seen with other protoparvovirus VLPs (16, 66). On the other hand, the absorbances from control rabbit sera and mouse mAb B7 were blocked with self-antigen (data not shown), revealing that the assay and blocking procedure worked as desired. Thus, no MVMp-specific immune reactivity could be detected by the two tested EIA methods. Additional serological techniques, such as indirect IF, western blot, or neutralization of infectivity, performed with control MVMp and permissive NB324K cells (see Materials and Methods) did not give reliable positive results and were further complicated by the heterogeneity of the backgrounds (data not shown). Hence, no convincing serological evidence for the presence of specific antibodies against MVMp in the tested human cohorts, with a total of 1,032 individuals, could be obtained.

In our second approach, we searched for the presence of MVMp genomes by simple or nested PCR at high sensitivity, as exemplified with control samples in Fig. S2. The genetic screen proceeded from total DNA isolated from peripheral blood or BM of a wide collection of samples from healthy individuals and patients with different hematopoietic pathologies, including immune deficiencies, different types of leukemias, myelodysplastic syndrome, and other severe hematopoietic dysfunctions, which could allow significant levels of MVMp growth. DNA samples for this screen are disclosed in three supplemental tables according to their origin: BM and peripheral blood from donors and onco-hematological patients subjected to BM transplantation (Table S4); BM from patients with a diagnosis of graft failure, poor graft function, and severe cytopenia, upon allogenic hematopoietic transplantation (Table S5); and hundreds of blood and plasma samples obtained in the African continent (Table S6) from a malaria endemicity research program in Ghana and DRC (68, 69). Despite the sensitive diagnostic procedure employed, in no case were significant and reproducible single or nested PCR amplifications obtained that would confidently indicate the presence of MVMp genomes in the samples. In conclusion, our epidemiological screens show no evidence of currently circulating MVMp in the tested human populations.

## DISCUSSION

### MVM and human hematopoiesis

To explore a putative natural tropism of MVMp toward humans, we first addressed its interaction with human hematopoietic progenitors in culture, using the genetically close MVMi strain as a control. This strain is a virulent mouse pathogen with a marked tropism for mouse hematopoiesis (37, 39, 43). Notably, the growth as colony-forming units of committed myeloid, erythroid, and megakaryocyte, as well as primitive CD34+ progenitors, is specifically inhibited by the MVMp infection (Fig. 1). The inhibitory capacity is observed on human hematopoietic cells from three different sources (umbilical cord blood, BM, or G-CSF-mobilized peripheral blood). The inhibition does require infectious MVMp viral particles and is not observed in parallel MVMi infections performed at similar MOI (Fig. 1 and 2). Importantly, the human progenitors committed to different hematopoietic lineages were similarly inhibited by infectious MVMp, which indicates that the virus uses a receptor shared by the hematopoietic lineages and primitive CD34+ cells to infect. It is intriguing that the closely related MVMi strain targets primitive mouse spleen colony-forming progenitors (37) and long-term repopulating hematopoietic stem cells (39), raising the question of the capacity of MVMp to similarly target genuine human hematopoietic stem cells. Future studies on MVMp infections in Long Term Culture Initiating Cells (70) and NOD/SCID repopulating assays (71) may help in solving this important question. In any case, the capacity of MVMp to cytotoxically infect primary human hematopoietic progenitors broadens the species specificity of the MVM parvovirus species to humans.

The inhibition of colony formation by the MVMp-infected human progenitors is likely caused by the expression of the cytotoxic viral non-structural protein NS-1 (2, 59, 60) observed in a high proportion of CD34+ progenitors in culture (Fig. 3B and C). These human hematopoietic cultures also support the synthesis of substantial levels of viral DNA replicative intermediates, but neither accumulation of ssDNA viral genomes (Fig. 3A) nor production of infectious mature viruses. An abortive viral infection may be related to cell differentiation, as the hematopoietic progenitors differentiate rapidly when cultured *in vitro*, changing their protein expression pattern (72, 73). It could be argued that the cell functions required to support the infectious progression of MVMp in human hematopoietic cells would be lost upon cell differentiation induced in the liquid cultures. In other words, the extinction of human hematopoietic cells susceptible to MVMp would take place during culturing. This phenomenon relates to the abortive MVMp infection in some established cell lines (74) and primary human cancer stem cells (50), whereas it may complete a productive life cycle in human-transformed cell lines of other origins (57–60). Interestingly, cell transformation modifies the genetic program toward a cellular state resembling primitiveness, and factors acting during development are also functional in tumor cells but not in their normal non-transformed counterparts (71–73). Therefore, hematopoietic precursors, regardless of the stemness of every particular niche, can be considered highly undifferentiated, a circumstance that could be at the core of the susceptibility to MVMp infection.

B19V of the genus *Erythroparvovirus,* so far the only other characterized parvovirus with the capacity to interfere with human hematopoiesis (14, 17–21), inhibits the colony formation by human precursors committed to the erythroid but not to the myeloid lineage (28, 29), and this inhibitory capacity increases with the differentiation stage from the BFU-E to the CFU-E precursor (30). This narrow tropism contrasts with that of the MVMp, whose toxic infection capacity for the CD34^+^ human primitive precursors results in the absence of clonogenic activity of precursors committed to both the erythroid (BFU-E to the CFU-E) and the myeloid (CFU-GM) lineages (Fig. 1 and 2). Nevertheless, both viruses share important characteristics in their life cycles in human hematopoietic cultures. B19V genome replicative intermediates can be amplified to high levels in differentiating erythroid precursors (30–32) coupled to the expression of the NS1 major viral replicative protein (75), as we find for MVMp in CD34+ progenitor cultures (Fig. 3A through C). Likewise, B19V infections of cultured human bone marrow cells yield minor amounts of infectious mature virus progeny (14, 75), paralleling the non-productive MVMp infection (Fig. 3). This suggests a concourse of poorly characterized mechanisms restricting the virus life cycle. A genome packaging defect identified in the B19V-UT7/Epo leukemic cell line interaction (76) may explain, at least in part, the nature of these restrictions and perhaps account for the failed increase in ssDNA viral genome accumulation found in MVMp-infected primary CD34+ cultures (Fig. 3A). Interestingly, despite these growth restrictions in culture, natural B19V infections may result in very high levels of viremia (14, 21), suggesting that the complexity of erythroid receptors and regulatory intracellular factors required for productive infection cannot be reproduced *in vitro*. This raises the intriguing possibility that putative MVMp infections of susceptible human patients might also lead to profound hematopoietic dysfunctions and elevated pathogenic viremia.

### Modeling MVMp interaction with human hematopoiesis at the organismal level

As a preliminary model to gain insights into MVMp’s suppressive capacity against human hematopoiesis *in vivo*, we compared MVM strains’ replication, evolution, pathogenicity, and hematopoietic impact over time in basal versus BM-humanized immunodeficient NSG mice. Although our genetic analysis focused on consensus Sanger sequences of virus populations, without approaching the biological significance of each mutation, it allowed us to identify amino acid changes in the capsid sbd critical for tropism determination. In basal NSG mice, MVMi replicates at high levels in hematopoietic organs and kills mice within 90 days (Fig. 5A and B), a time period similar to the lethal leukopenic process in the scid genetic background (43), without involving genetic evolution in the sbd (Table 1, lower). This analysis consistently supports MVMi as a natural virulent mouse pathogen with pan-hematopoietic tropism (37, 39, 43). In contrast, MVMp shows low pathogenicity and replicates at much lower levels in NSG mice (Fig. 5A and B), yet the accumulated viral genomes carry in some mice the G321E and S377N changes in the sbd (Table 1, upper), denoting genetic adaptation to mouse hematopoiesis for virus growth. These results are consistent with the inability of MVMp to infect mouse hematopoietic progenitors *in* vitro (37) and the long-term adaptation to the scid mouse hematopoiesis through changes at the sbd (56). The fact that the selected changes at the sbd do not overlap in the MVMp variants recovered from the scid and NSG mice may indicate a distinct adaptation to the types of sialic acid characteristics of the genetic background of each mouse strain (77). Interestingly, other selected capsid changes localize in a groove surrounding the 5× axis of symmetry of the capsid, as the MVMp-Q508L in NSG and the MVMi-I497M in all mice (albeit at lower frequency in BM-chimeric mice), the latter a highly heterogeneous position (33) commonly observed in MVM stocks grown in mouse lymphoid cells (34), suggesting that some interactions outside the sbd may be required to infect certain types of mouse cells. In summary, all our studies on hematopoietic interactions, together with many other experimental evidence *in vitro* and in mice referred above (see Introduction), strongly suggest that the mouse is not a natural host for MVMp.

Our attempts to support the MVMp tropism to human hematopoietic progenitors described above in the BM-humanized NSG mice system are made difficult as these animals maintain substantial levels of mouse hematopoiesis, as well as certain immunocompetence that drastically restrains virus multiplication. These complications are well illustrated by the decline of human CD34+ precursors in h/NSG mice (Fig. 4) under low levels of MVMi replication (Fig. 5A), as this virulent strain can cause indirect damage to overall BM functionality through a severe depletion of the supportive mouse hematopoiesis. Despite these limitations, under similarly restrained replication levels (Fig. 5A, right), the suppressive effect on human CD34+ precursors (Fig. 4B and C) and the impact on mouse survival (Fig. 5B, right) were more severe in MVMp than in MVMi infections. Further evidence came from the emergence of mutations at the sbd selected in h/NSG but not in basal NSG mice, suggesting adaptation to human hematopoiesis. That was the case for the V366L in MVMi and the A551V in MVMp infections (Table 1), although the extensive selection of the latter mutation in MVMp-infected scid mice (56) makes the host origin of the hematopoietic cells driving the selection uncertain. In summary, the genetic analysis of MVM capsids suggests particular adaptations of each strain to human or mouse hematopoiesis and further highlights their remarkable evolutionary capacity, enabling rapid host adaptation under compromised immune responses. These features may underlie a possible origin of MVMp with potential pathogenicity for humans from an MVMi-like mouse virus, an inter-species adaptive jump that may have occurred without an intermediate host, given the genetic proximity between the two strains.

### Searching for circulating MVM in human populations

The need to anticipate viral control in humans caused by unexpected viruses relies heavily on identifying the status of populations that are unaware of these potential infections, especially in developing countries. Hence, the hematopoietic evidence on MVMp as a putative human pathogen prompted us to run a large epidemiological survey using two complementary and sensitive diagnostic approaches. By serology, over 1,000 serum or plasma samples collected from healthy or ill individuals, including hematopoietic patients, from three countries (Finland, Iran, and Spain) screened by two EIA methods yielded no convincing serological evidence on the presence of specific antibodies against MVMp in these human cohorts (Table S3). In parallel, simple and highly sensitive nested PCR protocols failed to detect MVMp genomes in total DNA samples purified from (i) BM and/or peripheral blood of onco-hematopoietic patients subjected to BM transplants (Table S4), (ii) BM of immunosuppressed patients with a diagnosis of graft failure and severe cytopenia following allogenic hematopoietic transplantation (Table S5), and (iii) hundreds of individuals from Africa, belonging to a malaria endemicity research program (Table S6). Therefore, it is unlikely that MVMp could be circulating at high levels in the geographic regions where we conducted our epidemiological study.

However, despite our inability to detect anti-MVMp antibodies or genomes in the 1,924 human samples screened, we cannot discard that MVMp may be circulating as a human pathogen in other geographical areas, or at levels lower than the detection threshold of our survey, or persisting in tissues and organs distinct from the hematopoietic system, not addressed in this study. Temporary limitations should also be considered, and the possibility that MVMp circulated in the past but is currently an extinct human pathogen cannot be discarded. Some of these difficulties resemble those encountered when addressing the epidemiological surveillance of the human erythroparvovirus B19V, such as restricted growth *in vitro* (31, 32), persistence in tissues outside the BM (22–27), association with diseases not directly caused by dysfunctions of the erythroid lineage (21), and extinction of circulating genotypes (22, 23). Other related restrictions compromising the assignment of novel human parvoviruses have been previously discussed (15).

### Concluding remarks

We have demonstrated that MVMp, a virus classified as a mouse pathogen, can directly and cytotoxically infect human primitive CD34+ cells, as well as precursors committed to several hematopoietic lineages (Fig. 1 to 3). Furthermore, MVMp depresses human primitive progenitors in BM-humanized immunodeficient mice and, unlike MVMi, compromises the survival of transplanted mice without major genetic adaptation (Fig. 4 and 5). Interestingly, the tropism of MVMp toward human primitive and committed erythroid and myeloid hematopoietic progenitors tightly parallels that of MVMi to mouse hematopoiesis progenitors and long-term repopulating stem cells (37, 39, 41), suggesting that MVMp harbors the potential of being an air-borne human pathogen causing severe leukopenia and dysregulated erythropoiesis in immune-compromised patients, as MVMi does in intranasally inoculated immunodeficient mice (43). Although we were unable to find evidence of currently circulating MVMp in humans from different continents, its high potential as a human hematopoietic pathogen deserves further epidemiological searches, as well as a careful evaluation of prevalence and disease association in human populations.

## MATERIALS AND METHODS

### Hematopoietic cells and CD34^+^ purification

Mononuclear cells were obtained from umbilical cord blood scheduled for discard after a normal full-term delivery, following the informed consent of the mother. Mononuclear cells from peripheral blood (PB) and BM were obtained from healthy volunteers by Ficoll-Hypaque centrifugation. CD34^+^ cells were obtained from UCB MNCs by magnetic sorting using an anti-CD34 MicroBead Kit and positive immunomagnetic selection through LS columns (Miltenyi, Biotech SL, Spain) following the manufacturer’s instructions. Cell purity was analyzed by flow cytometry, and values > 95% were routinely obtained.

### *In vitro* hematopoietic colony assays and liquid cultures

An appropriate number of viable cells (0,5–1 × 10^5^ cells/mL for MNCs, and 1–3 × 10^3^ cells/mL for CD34^+^ cells) were seeded in dishes containing MethoCult GF H4434 culture media (StemCell Technologies, Vancouver, Canada) for evaluating the number of CFU-GM (myeloid progenitors) and BFU-E (erythroid progenitors) after 14 days of incubation. For the determination of CFU-MK (megakaryocytic progenitors), cells were grown for 11 days with Megacult C (StemCell Technologies). In all instances, cultures were maintained at 37°C in 5% CO_2_ and fully humidified air. For liquid cultures, cells were seeded in IMDM and 20% FBS supplemented with 10 ng/mL mIL3, 20 ng/mL hTPO, and 4 U/mL mSCF and maintained at 37°C in 5% CO_2_ and fully humidified air.

### Virus strains and cell infections

The sources of our MVMp and MVMi viral strains and infectious molecular clones have been previously described (50). The respective viral stocks were produced by electroporation of NB324K cells with the respective infectious molecular clones and subsequent propagation in cultures infected at low multiplicity (78). Viruses were purified by sucrose cushions and cesium chloride equilibrium centrifugation and kept at −70°C in aliquots following previously described procedures (64). Infectious titers were determined on the NB324K cell line by an optimized plaque-forming unit assay (79). Purified viral particles and empty capsids were quantitated by their hemagglutination activity with mouse erythrocytes at 4°C (64).

MNC or CD34^+^ cells were resuspended in IMDM with 2% FCS at a concentration of 1–2 × 10^6^ cells/mL and incubated with different multiplicities of infection for 1.5 hours at 37°C with constant shaking. For *in vitro* hematopoietic colony assays, four volumes of IMDM + 10% FBS were then added to the cells before seeding into semisolid media. For *in vitro* liquid culture, cells were washed in IMDM + 10% FBS to remove unbound viruses and were then seeded in IMDM + 20% FBS supplemented with 10 ng/mL mIL-3, 20 ng/mL hTPO, and 4 U/mL mSCF. Cell growth was followed by daily counting of viable cells using the Trypan Blue exclusion method.

### Virus gene expression and genome replication *in vitro*

To study NS-1 protein expression by flow cytometry, cells were washed with PAB (PBS^-^ 1× + 0.1% Na_3_N + 0.5% BSA) and fixed in 1% PFA at 4°C for 30 min. After washing with PAB, cells were permeabilized in PABT (PAB + 0.1% Triton X-100) for 3 min at 4°C and then a mouse anti-NS-1 monoclonal antibody (39) was added and incubated for 1 hour at 4°C. Cells were washed and stained with an anti-mouse-Biotin antibody and streptavidin-Tricolor (Caltag, South San Francisco, CA, USA) and analyzed in a Coulter EPICS XL (Coulter, Hialeah, FL, USA) flow cytometer. At least 10^4^ total events were collected per sample. Offline analysis was performed using the WinMDI free software package and FlowJo software (BD Biosciences, Spain).

For western blot analysis, 100 µg/well of soluble proteins from CD34^+^ cells infected with MVMp were separated by SDS-PAGE (7.5% polyacrylamide gels) and transferred to polyvinylidene difluoride membranes (Immobilon P; Millipore, Bedford, MA, USA) in 25 mM Tris, 190 mM glycine, and 5% methanol, pH 8.3. Membranes were blocked with 5% skimmed milk in PBS and then incubated for 1 hour with a 10^−4^ dilution of a rabbit anti-NS1 polyclonal antibody (64). Binding of the primary antibody was detected by incubation with secondary horse-radish peroxidase (HRP)-conjugated sheep anti-rabbit Ig Abs (in 2.5% skimmed milk in PBS) used at 10^−4^ dilution, followed by ECL detection (Amersham Bioscience, USA). For viral DNA replication analysis, CD34^+^ cells were collected at the indicated post-infection times and proceeded for low-molecular-weight DNA isolation with carrier tRNA to ensure quantitative yields as described (50). MVM genomic and intermediate replicative forms were resolved by agarose electrophoresis and developed by hybridization in a Southern blot method to a full-length ^32^P-labeled MVM-specific probe.

### Mice procedures

Non-obese diabetic immunodeficient NOD.Cg-*Prkdc^scid^Il2rg^tm1Wjl^*/ThomJ (NSG) mice (purchased from The Jackson Laboratory) were used. These mice carry the *scid* (DNA repair complex protein *Prkdc*) and the *IL2rg^null^* mutations on the NOD/ShiLtJ genetic background and are therefore extremely immunodeficient. Mice were housed and bred at the CIEMAT Laboratory Animals Facility (registration number ES280790000183), where they were routinely screened for pathogens in accordance with the Spanish Society for the Laboratory Animal Science and the Federation of European Laboratory Animal Science Associations (Tomworth, UK) recommendations, and no pathogens were found. Mice were provided with food (TEKLAD Global Diet 2918, irradiated with 25 KGy gamma rays) and water (acidified and autoclaved) *ad libitum* under controlled environmental conditions. Mice were housed during the experimental protocols in micro-insulators, individually ventilated cages type IIL with 25 air cage changes per hour. A maximum of six mice were housed in each cage. Room lighting was controlled with light/dark cycles of 13/11 hours, and temperature and humidity were regulated at 20°C ± 2°C and 55% ± 10%, respectively. All experimental procedures were conducted according to European and Spanish regulations: Directive 2009/41/CE and Spanish Law 9/2003 and R.D. 178/2004.

For BM humanization of the mice, human CD34^+^ cells were administered through the tail vein of 6–8-week-old female NSG mice sub-lethally irradiated with 1.5 Gy the day before transplant. Human engraftment analyses were conducted at periodic intervals after transplantation by BM aspiration from one femur by puncture through the knee joint, according to a previously described procedure (80). For infection, the mice were inoculated intravenously in the tail vein with 0.22 µm sterile-filtered purified viral stocks with a viral dose of 2.5 × 10^5^ PFU/mouse of MVMi and 2.0 × 10^7^ PFU/mouse of MVMp in 0.02 mL of phosphate-buffered saline (PBS). At the end of the experiments, mice were sacrificed, and bone marrow (harvested by crunching the bones), peripheral blood, and spleen were analyzed by flow cytometry (BD LSR Fortessa; BD Biosciences). Cells were stained with hCD45-APC-Cy7 (clone HI30, BioLegend), hCD19-PECy7 (clone SL25C1, BioLegend), hCD33-PE (Beckman Coulter), and hCD34-APC (clone 581, BioLegend). 4′,6-diamidino-2-phenylindole dihydrochloride-positive cells were excluded from the analysis. Analysis was performed using FlowJo software (BD Biosciences).

### Serology of human populations

For serology testing, virus-like particles engineered from the MVMp capsid in a recombinant baculovirus expression system (81) were produced in High5 insect cells as previously described (59). The VLP quality and the enzyme immune assay responses were systematically analyzed with an anti-VPs rabbit polyclonal serum (81) and the mAb-B7 mouse monoclonal antibody recognizing intact MVMp capsid (40, 82) under various antibody and substrate concentrations, along with assay conditions, to establish the following two EIA methods for the screening of human serum samples. In the direct immobilization of the antigen method, the VLPs (30 ng/well) were immobilized on 96-well plates (Costar 96-Well EIA/RIA Stripwell Plates, Corning) overnight at 4°C in PBS, emptied, and kept uncovered at 37°C for 30 min. In the streptavidin-linked biotinylated antigen method, the VLPs were biotinylated using the EZ-Link Sulfo-NHS-LC-Biotin kit (Thermo Fisher Scientific) and immobilized (40 ng/well) on streptavidin-coated plates (UniverSA96-Lockwell, Kaivogen) for 1 hour at room temperature under 400 rpm shaking in PBS-0.05% Tween 20 (PBST) (66). To minimize non-specific background, both plate types were post-coated 3× for 10 min with the Diluent (150 µL per well) (Labsystems Diagnostics). Next, human plasma or serum samples diluted 1:200 in RED buffer (Kaivogen) were applied and incubated for 1 hour at 37°C, followed by extensive washing with PBST. The EIAs were developed with HRP-conjugated anti-human IgG (DAKO) diluted 1:4,000 in diluent and 3,3′,5,5′-tetramethylbenzidine (BD OptEIA). The reaction was stopped with 0.5 M H_2_SO_4_, and the ODs were measured at 450 nm (Multiskan EX, Thermo Fisher Scientific). Samples showing measurable OD values (≥0.2) were subjected to homotypic- and heterotypic-VLP competition, as previously described (66). Additional serology screenings were performed with some human sera attempting to demonstrate anti-MVMp capsid protein immune reactivity by indirect immunofluorescence (82), western blot (64), and neutralization (44, 83), employing samples of MVMp-infected NB324K cells.

### Human hematopoietic sampling for PCR

Human DNA samples obtained from healthy and hematopoietic patients of different geographic distribution were from three sources: (i) peripheral blood and BM samples from anemic and onco-hematological patients from the Centro de Transfusión Comunidad de Madrid (Spain) (Table S4); (ii) 16 BM samples from onco-hematological patients treated by allogenic transplantation at the Hospital Fundación Jimenez Díaz (Madrid, Spain) (Table S5). Total DNA was prepared manually by the QIAamp DNA Blood Mini Kit; (iii) blood and plasma samples from African donors (Table S6) were preserved in dried blood spot (DBS) or dried serum spot (DSS) format that allows transport and storage at room temperature suitable for subsequent DNA extraction (84). From each individual, 2 mL of peripheral blood was collected by venipuncture, and 600 µL was preserved as dried blood, which was arranged as 12 × 50 µL DBS on Whatman FTA Classic Filter Paper Cards (GE Healthcare). The remaining blood was centrifuged to obtain plasma, so DSS was generated in an identical manner. All cards were air dried for 12 hours and stored at room temperature individually in desiccant-sealed bags. DNA from dried blood spots was extracted with the InstaGene Whole Blood Kit (Bio-Rad) following the modification previously described (84) and was used as a template for PCR.

### PCR and qPCR detection and sequencing of MVM genomes

Mice organs (spleen or flushed BM) were explanted and homogenized in PBS (10%, wt/vol) to isolate low-molecular-weight DNA following a modified Hirt procedure (39, 56). MVM replicative and genomic DNA forms were next enriched by phenol-chloroform and DNA purification by Mag-Bind TotalPure NGS (Omega Bio-Tek) using 0.7 volumes of beads. Viral genomes were quantified by real-time PCR (qPCR) using oligonucleotides targeting an NS gene region of 101 bp (Table S2) and the NZYSupreme qPCR Green Master Mix (NZYtech) according to the manufacturer’s instructions. These qPCRs were performed in a Bio-Rad CFX Opus 384 (Roche) system with a thermal protocol that consisted of an initial 5 min denaturation step at 95°C, followed by 40 cycles of two steps: denaturation at 95°C for 5 s and annealing-extension at 60°C for 25 s. Final melting curves were obtained by increasing the temperature from 65°C to 95°C (0.5°C every 5 s) to rule out unspecific amplifications. Linear regression of PCR cycle thresholds (Ct) obtained with a standard consisting of known amounts of a linearized infectious MVM plasmid showed *R*^2^ values above 0.98 and PCR efficiencies between 90% and 105% over a dynamic range of six log units. Technical triplicates were obtained for each sample, discarding those that showed a difference of one cycle compared to the other two.

Viral genetic screening of MVM in human samples was performed by nested PCR. Amplifications were undertaken with NZYTaq II 2× Green Master Mix (Nzytech) using the oligonucleotides outlined in Table S2. The PCRI (~1.8 Kbp of the VP gene) was performed with 1–15 µL of the human DNA samples in a final reaction volume of 50 µL, under cycling conditions consisting of an initial denaturation and polymerase activation at 95°C for 10 min, followed by 35 cycles at 94°C for 15 s, 56°C for 5 s, and 72°C for 30 s. Then, 2–4 µL of the PCRI product was used as template for two nested PCRs of different purposes in a final reaction volume of 25 µL. The PCRIIA targeting 423 bp of the VP gene was conducted for MVM genome screening. The thermal protocol was similar to that of the PCRI, with an annealing temperature of 58.5°C and 72°C extensions for 10 s. This protocol proved highly sensitive in detecting 10 molecules of MVM genomes (Fig. S2). The PCRIIB amplified 1,691 bp of the VP gene, and the products were subjected to DNA sequencing. In this case, the annealing was at 54°C and extensions at 72°C for 30 s. Any amplified DNA bands denoting putative MVM genomes were purified from agarose gels using a Wizard SV Gel and PCR Clean-Up System (Promega) and Sanger-sequenced with internal oligonucleotides (Unidad de Genómica CAI, UCM, Madrid, Spain). The obtained DNA sequences were analyzed by BLAST (https://blast.ncbi.nlm.nih.gov/Blast.cgi/) and Clustal Omega (https://www.ebi.ac.uk/Tools/msa/clustalo/).

### Statistical analysis

Hematopoietic data are represented as mean ± standard error of the mean. The significance of differences was determined by the non-parametric Wilcoxon’s Mann-Whitney *W*-test, and multiple comparisons were determined by the *F*-test ANOVA using Statgraphics Plus 5.0 software package (Manugistics Inc., Rockville, MD, USA). Statistical analysis of the imaging studies was performed using GraphPad Prism software. The statistical significance of qPCR assays from h/NSG samples was determined by an unpaired *t* test 95% confidence interval, with Welch’s correction, upon confirmed data normality by Shapiro-Wilks. The mutation percentage in virus genomes was determined by measuring the respective area of each nucleotide at a precise position of the sequence profiles by Fiji (ImageJ).

## Data Availability

The authors confirm that the data supporting the findings of this study are available within the article and its supplemental materials. Any additional information will be made available on request.

## SUPPLEMENTAL MATERIAL

Supplemental material spectrum.02339-25-s0001.docx Figures S1 and S2; Tables S1 to S6.

ASM does not own the copyrights to Supplemental Material that may be linked to, or accessed through, an article. The authors have granted ASM a non-exclusive, world-wide license to publish the Supplemental Material files. Please contact the corresponding author directly for reuse.

## References

[1] Pénzes JJ, Söderlund-Venermo M, Canuti M, Eis-Hübinger AM, Hughes J, Cotmore SF, Harrach B. 2020. Reorganizing the family Parvoviridae: a revised taxonomy independent of the canonical approach based on host association. Arch Virol 165:2133–2146. doi:10.1007/s00705-020-04632-4 32533329

[2] Cotmore SF, Agbandje-McKenna M, Canuti M, Chiorini JA, Eis-Hubinger A-M, Hughes J, Mietzsch M, Modha S, Ogliastro M, Pénzes JJ, Pintel DJ, Qiu J, Soderlund-Venermo M, Tattersall P, Tijssen P, Ictv Report Consortium. 2019. ICTV virus taxonomy profile: parvoviridae. J Gen Virol 100:367–368. doi:10.1099/jgv.0.001212 30672729 PMC6537627

[3] Shackelton LA, Parrish CR, Truyen U, Holmes EC. 2005. High rate of viral evolution associated with the emergence of carnivore parvovirus. Proc Natl Acad Sci USA 102:379–384. doi:10.1073/pnas.0406765102 15626758 PMC544290

[4] François S, Filloux D, Roumagnac P, Bigot D, Gayral P, Martin DP, Froissart R, Ogliastro M. 2016. Discovery of parvovirus-related sequences in an unexpected broad range of animals. Sci Rep 6:30880. doi:10.1038/srep30880 27600734 PMC5013282

[5] He WT, Hou X, Zhao J, Sun J, He H, Si W, Wang J, Jiang Z, Yan Z, Xing G, Lu M, Suchard MA, Ji X, Gong W, He B, Li J, Lemey P, Guo D, Tu C, Holmes EC, Shi M, Su S. 2022. Virome characterization of game animals in China reveals a spectrum of emerging pathogens. Cell 185:1117–1129. doi:10.1016/j.cell.2022.02.014 35298912 PMC9942426

[6] Bashir T, Horlein R, Rommelaere J, Willwand K. 2000. Cyclin A activates the DNA polymerase delta -dependent elongation machinery in vitro: a parvovirus DNA replication model. Proc Natl Acad Sci USA 97:5522–5527. doi:10.1073/pnas.090485297 10792046 PMC25861

[7] Weitzman MD. 2006. The parvovirus life cycle: an introduction to molecular interactions important for infection, p 143–156. In Kerr JR (ed), Parvoviruses. Hodder Arnold, London.

[8] Gil-Ranedo J, Hernando E, Riolobos L, Domínguez C, Kann M, Almendral JM. 2015. The mammalian cell cycle regulates parvovirus nuclear capsid assembly. PLoS Pathog 11:e1004920. doi:10.1371/journal.ppat.1004920 26067441 PMC4466232

[9] Abrahams RR, Majumder K. 2025. Small genomes, big disruptions: parvoviruses and the DNA damage response. Viruses 17:494. doi:10.3390/v17040494 40284937 PMC12031541

[10] Siegel G. 1988. Patterns of parvovirus disease in animals, p 43–67. In Pattison JR (ed), Parvoviruses and human disease. CRC Press, Boca Raton, FL.

[11] Kurtzman GJ, Platanias L, Lustig L, Frickhofen N, Young NS. 1989. Feline parvovirus propagates in cat bone marrow cultures and inhibits hematopoietic colony formation in vitro. Blood 74:71–81. doi:10.1182/blood.V74.1.71.71 2546625

[12] Brown KE, Anderson SM, Young NS. 1993. Erythrocyte P antigen: cellular receptor for B19 parvovirus. Science 262:114–117. doi:10.1126/science.8211117 8211117

[13] Allander T, Tammi MT, Eriksson M, Bjerkner A, Tiveljung-Lindell A, Andersson B. 2005. Cloning of a human parvovirus by molecular screening of respiratory tract samples. Proc Natl Acad Sci USA 102:12891–12896. doi:10.1073/pnas.0504666102 16118271 PMC1200281

[14] Qiu J, Söderlund-Venermo M, Young NS. 2017. Human parvoviruses. Clin Microbiol Rev 30:43–113. doi:10.1128/CMR.00040-16 27806994 PMC5217800

[15] Söderlund-Venermo M. 2019. Emerging human parvoviruses: the rocky road to fame. Annu Rev Virol 6:71–91. doi:10.1146/annurev-virology-092818-015803 31283445

[16] Chesnut SK, Mohanraj U, Rayamajhi Thapa R, Jalilian FA, Amini R, Sedighi I, Sedighi P, Al-Hello H, Barakat AM, Masika M, Mwaengo D, Anzala O, Nora-Krukle Z, Vilmane A, Ziemele I, Manaresi E, Gallinella G, Viikari L, Jartti T, Söderlund-Venermo M. 2025. In search of human protoparvovirus acute infections. Virology (Auckl) 608:110529. doi:10.1016/j.virol.2025.110529 40233444

[17] Heegaard ED, Brown KE. 2002. Human parvovirus B19. Clin Microbiol Rev 15:485–505. doi:10.1128/CMR.15.3.485-505.2002 12097253 PMC118081

[18] Smith-Whitley K, Zhao H, Hodinka RL, Kwiatkowski J, Cecil R, Cecil T, Cnaan A, Ohene-Frempong K. 2004. Epidemiology of human parvovirus B19 in children with sickle cell disease. Blood 103:422–427. doi:10.1182/blood-2003-01-0069 14525777

[19] Pattison JR, Jones SE, Hodgson J, Davis LR, White JM, Stroud CE, Murtaza L. 1981. Parvovirus infections and hypoplastic crisis in sickle-cell anaemia. The Lancet 317:664–665. doi:10.1016/S0140-6736(81)91579-8 6110886

[20] Young NS, Mortimer PP, Moore JG, Humphries RK. 1984. Characterization of a virus that causes transient aplastic crisis. J Clin Invest 73:224–230. doi:10.1172/JCI111195 6317715 PMC425004

[21] Young NS, Brown KE. 2004. Parvovirus B19. N Engl J Med 350:586–597. doi:10.1056/NEJMra030840 14762186

[22] Söderlund M, von Essen R, Haapasaari J, Kiistala U, Kiviluoto O, Hedman K. 1997. Persistence of parvovirus B19 DNA in synovial membranes of young patients with and without chronic arthropathy. The Lancet 349:1063–1065. doi:10.1016/S0140-6736(96)09110-6 9107245

[23] Norja P, Hokynar K, Aaltonen L-M, Chen R, Ranki A, Partio EK, Kiviluoto O, Davidkin I, Leivo T, Eis-Hübinger AM, Schneider B, Fischer H-P, Tolba R, Vapalahti O, Vaheri A, Söderlund-Venermo M, Hedman K. 2006. Bioportfolio: lifelong persistence of variant and prototypic erythrovirus DNA genomes in human tissue. Proc Natl Acad Sci USA 103:7450–7453. doi:10.1073/pnas.0602259103 16651522 PMC1464359

[24] Pyöriä L, Toppinen M, Mäntylä E, Hedman L, Aaltonen LM, Vihinen-Ranta M, Ilmarinen T, Söderlund-Venermo M, Hedman K, Perdomo MF. 2017. Extinct type of human parvovirus B19 persists in tonsillar B cells. Nat Commun 8:14930. doi:10.1038/ncomms14930 28374737 PMC5382274

[25] Cassinotti P, Burtonboy G, Fopp M, Siegl G. 1997. Evidence for persistence of human parvovirus B19 DNA in bone marrow. J Med Virol 53:229–232. doi:10.1002/(SICI)1096-9071(199711)53:3<229::AID-JMV8>3.0.CO;2-A 9365887

[26] Adamson-Small LA, Ignatovich IV, Laemmerhirt MG, Hobbs JA. 2014. Persistent parvovirus B19 infection in non-erythroid tissues: possible role in the inflammatory and disease process. Virus Res 190:8–16. doi:10.1016/j.virusres.2014.06.017 24998884

[27] Xu M, Leskinen K, Gritti T, Groma V, Arola J, Lepistö A, Sipponen T, Saavalainen P, Söderlund-Venermo M. 2022. Prevalence, cell tropism, and clinical impact of human parvovirus persistence in adenomatous, cancerous, inflamed, and healthy intestinal mucosa. Front Microbiol 13:914181. doi:10.3389/fmicb.2022.914181 35685923 PMC9171052

[28] Mortimer PP, Humphries RK, Moore JG, Purcell RH, Young NS. 1983. A human parvovirus-like virus inhibits haematopoietic colony formation in vitro. Nature 302:426–429. doi:10.1038/302426a0 6835376

[29] Srivastava A, Lu L. 1988. Replication of B19 parvovirus in highly enriched hematopoietic progenitor cells from normal human bone marrow. J Virol 62:3059–3063. doi:10.1128/JVI.62.8.3059-3063.1988 3392774 PMC253750

[30] Takahashi T, Ozawa K, Takahashi K, Asano S, Takaku F. 1990. Susceptibility of human erythropoietic cells to B19 parvovirus in vitro increases with differentiation. Blood 75:603–610. doi:10.1182/blood.V75.3.603.603 2404522

[31] Ozawa K, Kurtzman G, Young N. 1986. Replication of the B19 parvovirus in human bone marrow cell cultures. Science 233:883–886. doi:10.1126/science.3738514 3738514

[32] Ozawa K, Kurtzman G, Young N. 1987. Productive infection by B19 parvovirus of human erythroid bone marrow cells in vitro. Blood 70:384–391. doi:10.1182/blood.V70.2.384.384 3038211

[33] Astell CR, Gardiner EM, Tattersall P. 1986. DNA sequence of the lymphotropic variant of minute virus of mice, MVM(i), and comparison with the DNA sequence of the fibrotropic prototype strain. J Virol 57:656–669. doi:10.1128/JVI.57.2.656-669.1986 3502703 PMC252781

[34] Sahli R, McMaster GK, Hirt B. 1985. DNA sequence comparison between two tissue-specific variants of the autonomous parvovirus, minute virus of mice. Nucleic Acids Res 13:3617–3633. doi:10.1093/nar/13.10.3617 3855242 PMC341262

[35] Bonnard GD, Manders EK, Campbell DA Jr, Herberman RB, Collins MJ Jr. 1976. Immunosuppressive activity of a subline of the mouse EL-4 lymphoma. Evidence for minute virus of mice causing the inhibition. J Exp Med 143:187–205. doi:10.1084/jem.143.1.187 1244418 PMC2190089

[36] Engers HD, Louis JA, Zubler RH, Hirt B. 1981. Inhibition of T cell-mediated functions by MVM(i), a parvovirus closely related to minute virus of mice. J Immunol 127:2280–2285. doi:10.4049/jimmunol.127.6.2280 6457871

[37] Segovia JC, Real A, Bueren JA, Almendral JM. 1991. In vitro myelosuppressive effects of the parvovirus minute virus of mice (MVMi) on hematopoietic stem and committed progenitor cells. Blood 77:980–988. doi:10.1182/blood.V77.5.980.bloodjournal775980 1847313

[38] Lamana ML, Albella B, Bueren JA, Segovia JC. 2001. In vitro and in vivo susceptibility of mouse megakaryocytic progenitors to strain i of parvovirus minute virus of mice. Exp Hematol 29:1303–1309. doi:10.1016/S0301-472X(01)00724-X 11698126

[39] Segovia JC, Guenechea G, Gallego JM, Almendral JM, Bueren JA. 2003. Parvovirus infection suppresses long-term repopulating hematopoietic stem cells. J Virol 77:8495–8503. doi:10.1128/jvi.77.15.8495-8503.2003 12857918 PMC165232

[40] Ramírez JC, Fairén A, Almendral JM. 1996. Parvovirus minute virus of mice strain i multiplication and pathogenesis in the newborn mouse brain are restricted to proliferative areas and to migratory cerebellar young neurons. J Virol 70:8109–8116. doi:10.1128/JVI.70.11.8109-8116.1996 8892936 PMC190885

[41] Segovia JC, Bueren JA, Almendral JM. 1995. Myeloid depression follows infection of susceptible newborn mice with the parvovirus minute virus of mice (strain i). J Virol 69:3229–3232. doi:10.1128/JVI.69.5.3229-3232.1995 7707557 PMC189031

[42] Brownstein DG, Smith AL, Jacoby RO, Johnson EA, Hansen G, Tattersall P. 1991. Pathogenesis of infection with a virulent allotropic variant of minute virus of mice and regulation by host genotype. Lab Invest 65:357–364. 1653878

[43] Segovia JC, Gallego JM, Bueren JA, Almendral JM. 1999. Severe leukopenia and dysregulated erythropoiesis in SCID mice persistently infected with the parvovirus minute virus of mice. J Virol 73:1774–1784. doi:10.1128/JVI.73.3.1774-1784.1999 9971754 PMC104416

[44] López-Bueno A, Mateu MG, Almendral JM. 2003. High mutant frequency in populations of a DNA virus allows evasion from antibody therapy in an immunodeficient host. J Virol 77:2701–2708. doi:10.1128/jvi.77.4.2701-2708.2003 12552010 PMC141124

[45] López-Bueno A, Valle N, Gallego JM, Pérez J, Almendral JM. 2004. Enhanced cytoplasmic sequestration of the nuclear export receptor CRM1 by NS2 mutations developed in the host regulates parvovirus fitness. J Virol 78:10674–10684. doi:10.1128/JVI.78.19.10674-10684.2004 15367634 PMC516389

[46] Besselsen DG, Romero MJ, Wagner AM, Henderson KS, Livingston RS. 2006. Identification of novel murine parvovirus strains by epidemiological analysis of naturally infected mice. J Gen Virol 87:1543–1556. doi:10.1099/vir.0.81547-0 16690918

[47] Brownlee RD, Ardeshir A, Becker MD, Wagner AM, Besselsen DG. 2018. A field strain of minute virus of mice (MVMm) exhibits age- and strain-specific pathogenesis. J Gen Virol 99:558–566. doi:10.1099/jgv.0.001044 29517477 PMC5982129

[48] Crawford LV. 1966. A minute virus of mice. Virology (Auckl) 29:605–612. doi:10.1016/0042-6822(66)90284-4 5945715

[49] Tattersall P, Bratton J. 1983. Reciprocal productive and restrictive virus-cell interactions of immunosuppressive and prototype strains of minute virus of mice. J Virol 46:944–955. doi:10.1128/jvi.46.3.944-955.1983 6602222 PMC256569

[50] Gil-Ranedo J, Gallego-García C, Almendral JM. 2021. Viral targeting of glioblastoma stem cells with patient-specific genetic and post-translational p53 deregulations. Cell Rep 36:109673. doi:10.1016/j.celrep.2021.109673 34496248

[51] Ventoso I, Berlanga JJ, Almendral JM. 2010. Translation control by protein kinase R restricts minute virus of mice infection: role in parvovirus oncolysis. J Virol 84:5043–5051. doi:10.1128/JVI.02188-09 20219905 PMC2863853

[52] Hartley JW, Rowe WP. 1960. A new mouse virus apparently related to the adenovirus group. Virology (Auckl) 11:645–647. doi:10.1016/0042-6822(60)90109-4 14400125

[53] Geoffroy M-C, Salvetti A. 2005. Helper functions required for wild type and recombinant adeno-associated virus growth. Curr Gene Ther 5:265–271. doi:10.2174/1566523054064977 15975004

[54] Kimsey PB, Engers HD, Hirt B, Jongeneel CV. 1986. Pathogenicity of fibroblast- and lymphocyte-specific variants of minute virus of mice. J Virol 59:8–13. doi:10.1128/JVI.59.1.8-13.1986 3712557 PMC253031

[55] López-Bueno A, Rubio M-P, Bryant N, McKenna R, Agbandje-McKenna M, Almendral JM. 2006. Host-selected amino acid changes at the sialic acid binding pocket of the parvovirus capsid modulate cell binding affinity and determine virulence. J Virol 80:1563–1573. doi:10.1128/JVI.80.3.1563-1573.2006 16415031 PMC1346950

[56] López-Bueno A, Segovia JC, Bueren JA, O’Sullivan MG, Wang F, Tattersall P, Almendral JM. 2008. Evolution to pathogenicity of the parvovirus minute virus of mice in immunodeficient mice involves genetic heterogeneity at the capsid domain that determines tropism. J Virol 82:1195–1203. doi:10.1128/JVI.01692-07 18045943 PMC2224436

[57] Mousset S, Rommelaere J. 1982. Minute virus of mice inhibits cell transformation by simian virus 40. Nature 300:537–539. doi:10.1038/300537a0 6292735

[58] Rubio M-P, Guerra S, Almendral JM. 2001. Genome replication and postencapsidation functions mapping to the nonstructural gene restrict the host range of a murine parvovirus in human cells. J Virol 75:11573–11582. doi:10.1128/JVI.75.23.11573-11582.2001 11689639 PMC114744

[59] Caillet-Fauquet P, Perros M, Brandenburger A, Spegelaere P, Rommelaere J. 1990. Programmed killing of human cells by means of an inducible clone of parvoviral genes encoding non-structural proteins. EMBO J 9:2989–2995. doi:10.1002/j.1460-2075.1990.tb07491.x 2167840 PMC552016

[60] Mousset S, Ouadrhiri Y, Caillet-Fauquet P, Rommelaere J. 1994. The cytotoxicity of the autonomous parvovirus minute virus of mice nonstructural proteins in FR3T3 rat cells depends on oncogene expression. J Virol 68:6446–6453. doi:10.1128/JVI.68.10.6446-6453.1994 8083981 PMC237064

[61] Rubio M-P, López-Bueno A, Almendral JM. 2005. Virulent variants emerging in mice infected with the apathogenic prototype strain of the parvovirus minute virus of mice exhibit a capsid with low avidity for a primary receptor. J Virol 79:11280–11290. doi:10.1128/JVI.79.17.11280-11290.2005 16103180 PMC1193584

[62] Agbandje-McKenna M, Llamas-Saiz AL, Wang F, Tattersall P, Rossmann MG. 1998. Functional implications of the structure of the murine parvovirus, minute virus of mice. Structure 6:1369–1381. doi:10.1016/s0969-2126(98)00137-3 9817841

[63] Kontou M, Govindasamy L, Nam H-J, Bryant N, Llamas-Saiz AL, Foces-Foces C, Hernando E, Rubio M-P, McKenna R, Almendral JM, Agbandje-McKenna M. 2005. Structural determinants of tissue tropism and in vivo pathogenicity for the parvovirus minute virus of mice. J Virol 79:10931–10943. doi:10.1128/JVI.79.17.10931-10943.2005 16103145 PMC1193591

[64] Calvo-López T, Grueso E, Sánchez-Martínez C, Almendral JM. 2022. Intracellular virion traffic to the endosome driven by cell type specific sialic acid receptors determines parvovirus tropism. Front Microbiol 13:1063706. doi:10.3389/fmicb.2022.1063706 36756201 PMC9899843

[65] Ball-Goodrich LJ, Tattersall P. 1992. Two amino acid substitutions within the capsid are coordinately required for acquisition of fibrotropism by the lymphotropic strain of minute virus of mice. J Virol 66:3415–3423. doi:10.1128/JVI.66.6.3415-3423.1992 1316457 PMC241122

[66] Väisänen E, Paloniemi M, Kuisma I, Lithovius V, Kumar A, Franssila R, Ahmed K, Delwart E, Vesikari T, Hedman K, Söderlund-Venermo M. 2018. Epidemiology of two human protoparvoviruses, bufavirus and tusavirus. Sci Rep 6:39267. doi:10.1038/srep39267 PMC5155296 27966636

[67] Väisänen E, Mohanraj U, Kinnunen PM, Jokelainen P, Al-Hello H, Barakat AM, Sadeghi M, Jalilian FA, Majlesi A, Masika M, Mwaengo D, Anzala O, Delwart E, Vapalahti O, Hedman K, Söderlund-Venermo M. 2018. Global distribution of human protoparvoviruses. Emerg Infect Dis 24:1292–1299. doi:10.3201/eid2407.172128 29912685 PMC6038761

[68] Abad P, Marín-García P, Heras M, Fobil JN, Hutchful AG, Diez A, Puyet A, Reyes-Palomares A, Azcárate IG, Bautista JM. 2022. Microscopic and submicroscopic infection by Plasmodium falciparum: immunoglobulin M and A profiles as markers of intensity and exposure. Front Cell Infect Microbiol 12:934321. doi:10.3389/fcimb.2022.934321 36118030 PMC9478039

[69] Abad P, Coronado M, Vincelle-Nieto Á, Pérez-Benavente S, Fobil JN, Puyet A, Diez A, Reyes-Palomares A, Azcárate IG, Bautista JM. 2024. Shotgun characterization of the circulating IgM antigenome of an infectious pathogen by immunocapture-LC-MS/MS from dried serum spots. J Proteome Res 23:633–643. doi:10.1021/acs.jproteome.3c00439 38183416

[70] Szilvassy SJ, Humphries RK, Lansdorp PM, Eaves AC, Eaves CJ. 1990. Quantitative assay for totipotent reconstituting hematopoietic stem cells by a competitive repopulation strategy. Proc Natl Acad Sci USA 87:8736–8740. doi:10.1073/pnas.87.22.8736 2247442 PMC55034

[71] Larochelle A, Vormoor J, Hanenberg H, Wang JC, Bhatia M, Lapidot T, Moritz T, Murdoch B, Xiao XL, Kato I, Williams DA, Dick JE. 1996. Identification of primitive human hematopoietic cells capable of repopulating NOD/SCID mouse bone marrow: implications for gene therapy. Nat Med 2:1329–1337. doi:10.1038/nm1296-1329 8946831

[72] Ivanova NB, Dimos JT, Schaniel C, Hackney JA, Moore KA, Lemischka IR. 2002. A stem cell molecular signature. Science 298:601–604. doi:10.1126/science.1073823 12228721

[73] Zhao Y, Chen E, Li L, Gong B, Xie W, Nanji S, Dubé ID, Hough MR. 2004. Gene expression profiling in the inductive human hematopoietic microenvironment. Biochem Biophys Res Commun 323:703–711. doi:10.1016/j.bbrc.2004.08.140 15369807

[74] Guetta E, Graziani Y, Tal J. 1986. Suppression of Ehrlich ascites tumors in mice by minute virus of mice. J Natl Cancer Inst 76:1177–1180. doi:10.1093/jnci/76.6.1177 3458953

[75] Wong S, Zhi N, Filippone C, Keyvanfar K, Kajigaya S, Brown KE, Young NS. 2008. Ex vivo-generated CD36+ erythroid progenitors are highly permissive to human parvovirus B19 replication. J Virol 82:2470–2476. doi:10.1128/JVI.02247-07 18160440 PMC2258946

[76] Wolfisberg R, Ruprecht N, Kempf C, Ros C. 2013. Impaired genome encapsidation restricts the in vitro propagation of human parvovirus B19. J Virol Methods 193:215–225. doi:10.1016/j.jviromet.2013.06.003 23764418

[77] Dagur RS, Branch-Woods A, Mathews S, Joshi PS, Quadros RM, Harms DW, Cheng Y, Miles SM, Pirruccello SJ, Gurumurthy CB, Gorantla S, Poluektova LY. 2019. Human-like NSG mouse glycoproteins sialylation pattern changes the phenotype of human lymphocytes and sensitivity to HIV-1 infection. BMC Immunol 20:2. doi:10.1186/s12865-018-0279-3 30616506 PMC6322283

[78] Lombardo E, Ramírez JC, Garcia J, Almendral JM. 2002. Complementary roles of multiple nuclear targeting signals in the capsid proteins of the parvovirus minute virus of mice during assembly and onset of infection. J Virol 76:7049–7059. doi:10.1128/jvi.76.14.7049-7059.2002 12072505 PMC136310

[79] Gil-Ranedo J, Hernando E, Valle N, Riolobos L, Maroto B, Almendral JM. 2018. Differential phosphorylation and n-terminal configuration of capsid subunits in parvovirus assembly and viral trafficking. Virology (Auckl) 518:184–194. doi:10.1016/j.virol.2018.02.018 29524834

[80] Albella B, Segovia JC, Guenechea G, Pragnell IB, Bueren JA. 1999. Preserved long-term repopulation and differentiation properties of hematopoietic grafts subjected to ex vivo expansion with stem cell factor and interleukin 11. Transplantation 67:1348–1357. doi:10.1097/00007890-199905270-00010 10360589

[81] Hernando E, Llamas-Saiz AL, Foces-Foces C, McKenna R, Portman I, Agbandje-McKenna M, Almendral JM. 2000. Biochemical and physical characterization of parvovirus minute virus of mice virus-like particles. Virology (Auckl) 267:299–309. doi:10.1006/viro.1999.0123 10662625

[82] Grueso E, Sánchez-Martínez C, Calvo-López T, de Miguel FJ, Blanco-Menéndez N, Fernandez-Estevez M, Elizalde M, Sanchez J, Kourani O, Martin D, Tato A, Guerra M, Andrés G, Almendral JM. 2019. Antiangiogenic vascular endothelial growth factor-blocking peptides displayed on the capsid of an infectious oncolytic parvovirus: assembly and immune interactions. J Virol 93:e00798-19. doi:10.1128/JVI.00798-19 31315994 PMC6744233

[83] Kaufmann B, López-Bueno A, Mateu MG, Chipman PR, Nelson CDS, Parrish CR, Almendral JM, Rossmann MG. 2007. Minute virus of mice, a parvovirus, in complex with the Fab fragment of a neutralizing monoclonal antibody. J Virol 81:9851–9858. doi:10.1128/JVI.00775-07 17626084 PMC2045413

[84] Chaorattanakawee S, Natalang O, Hananantachai H, Nacher M, Brockman A, Krudsood S, Looareesuwan S, Patarapotikul J. 2003. Storage duration and polymerase chain reaction detection of Plasmodium falciparum from blood spots on filter paper. Am J Trop Med Hyg 69:42–44. doi:10.4269/ajtmh.2003.69.42 12932095

