# Supplementary material for: A presumed mouse parvovirus with overlooked high toxicity for human primitive CD34+ hematopoietic precursors in vitro and in bone marrow-humanized mice
Source: Microbiol Spectr. 2025 Oct 27;13(12):e02339-25. doi: 10.1128/spectrum.02339-25 (PMC12671093; doi:10.1128/spectrum.02339-25)
Supplement: Supplemental material — Figures S1 and S2; Tables S1 to S6. [file spectrum.02339-25-s0001.docx]

**
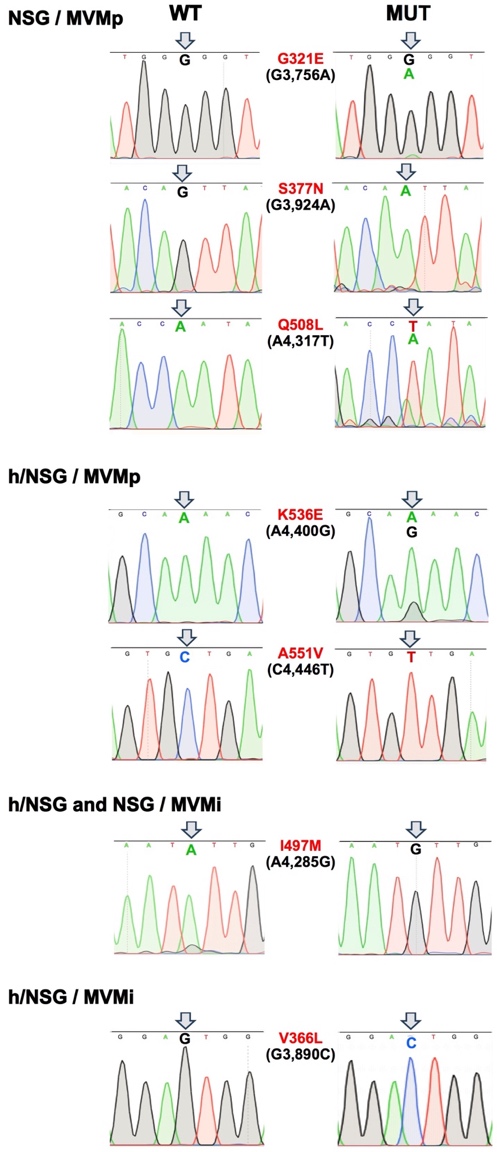
**

**Figure S1.** Representative sequence chromatograms at mutated positions and determined amino acid change in MVMp (*upper*) and MVMi *(lower*) infections of basal and BM-humanized NSG mice are illustrated. For comparison, the *wild-type* (WT) sequences are shown at the left and the consensus sequences found in mice organs (MUT) at the right. Numbers in bold indicate the mutations, and the corresponding VP2 amino acid changes are shown in red.

**
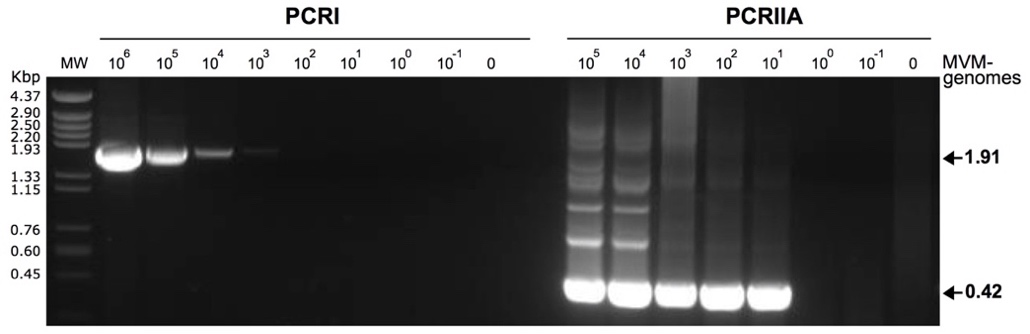
**

**Figure S2.** Sensitivity of PCR and Nested-PCR for the detection of MVM genomes. The figure shows an example of simple PCR (PCRI) or nested-PCR (PCRIIA) products resolved by agarose gel. MVM genomes used as templates were previously quantified by absolute qPCR and the amount used for each PCR is indicated above the gel lanes. The genome of phage phi-29 digested by *HindIII* was loaded as a molecular marker.

**Table S1.** PCR-Sanger detection of coding mutations selected in mice at the MVMp/i capsid genes.

| **NSG** | | | | | |
| --- | --- | --- | --- | --- | --- |
| Mutation | Aminoacid change^a^ | Virus | Times sequenced^b^ | Number of mice^c^ | Organ^d^ |
| G3756A | G321E | MVMp | 3 | 1 | 1 BMp^e^ |
| G3924A | S377N | MVMp | 7 | 2 | 2 BMp^e^, 1 BM^e^, 1 S |
| A4317T | Q508L | MVMp | 5 | 1 | 1 BMp, 1 BM^e^, 1 S |
| A4285G | I497M | MVMi | 6 | 4 | 4 BM, 2 S |
| **h/NSG** | | | | | |
| Mutation | Aminoacid change^a^ | Virus | Times sequenced^b^ | Number of mice^c^ | Organ^d^ |
| A4400G | K536E | MVMp | 2 | 1 | 1 BMp |
| C4446T | A551V | MVMp | 2 | 6 | 2 BM |
| G3890C | V366L | MVMi | 5 | 1 | 1 BMp, 1 BM^e^, 1 S |
| A4285G | I497M | MVMi | 4 | 4 | 1 BMp, 3 BM |

^a^Aminoacid change at VP2 numbering; ^b^independent Sanger-sequences of PCR products supporting the mutation; ^c^number of mice in which the mutation has been observed; ^d^BMp, Bone Marrow puncture; BM, Bone Marrow; S, spleen. ^e^at least two different extractions

**Table S2.** Primers targeting the MVM genome used in the study.

| **Primer name** | **Sequence (5´-3´)** | **Tm (ºC)** | **Position** | **Purpose** |
| --- | --- | --- | --- | --- |
| VVP3 | CGCACTAGACCACCTGC | 56 | 2.705-2.721 | PCRI and sequencing |
| VPSeq1CAT | CATGTTTCCAGTAGCAGTTGG | 62 | 4.510-4.490 | PCRI and sequencing |
| VVP7 | CCGTCAACTTGGACAGC | 54 | 3.652-3.668 | PCRIIA and sequencing |
| VPSeq2 | CCATTTAGTGGTGGTGG | 52 | 4.074-4.058 | PCRIIA and sequencing |
| NestedII-F | GCACAGCAAAGCAGTCAAAC | 57 | 2.774-2.793 | PCRIIB and sequencing |
| NestedII-R | GAGTTGCCATTGTCTTCAACACTT | 57 | 4.464-4.441 | PCRIIB and sequencing |
| NS1-F-qPCR | TAAGCGCGGCAGAATTCAAA | 58 | 1.074-1.093 | qPCR |
| NS1-R-qPCR | GGCTGCATCATCATCCAGTC | 60 | 1.181-1.162 | qPCR |
| VVP9 | CTCAAGGACAAATATGGGAC | 58 | 4.185-4.204 | sequencing |
| VVP6 | TGACAGAGATCTTTCAG | 48 | 3.445-3.461 | sequencing |

**Table S3.** Characteristics of the cohorts used in this study.

| Cohort | N | Health status | Median age (range) | EIA method | MVM IgG |
| --- | --- | --- | --- | --- | --- |
| Finland | 508 | Constitutionally healthy* | 37 (19-79) | dEIA | All neg |
| Iran | 169 | Constitutionally healthy and chronic** | 47 (18-86) | dEIA or sEIA | All neg |
| Iran | 335 | Healthy and diseased children*** | 2.2 (0.1-15) | sEIA | All neg |
| Spain | 20 | Adults**** | 40 (2-64) | dEIA or sEIA | All neg |

*Comprised 185 Helsinki University medical students and staff members as well as 323 participants at the national Annual Veterinary Congress in 2009 in Helsinki, 70% of whom reported contact with small rodents (66, 67). **Serum samples from 169 adult blood donors obtained at the Hamadan Blood Transfusion Organization (Hamadan, Iran) including 71 constitutionally healthy and 98 with chronic conditions, like diabetes or hyperlipidemia, but with no acute illnesses (67, 16). ***Serum samples from 9 healthy and 326 diseased children with mostly fever with unknown etiology, respiratory symptoms, and gastroenteritis (16). Obtained at the Hamadan Blood Transfusion Organization (Hamadan, Iran). ****Onco-hematological patients outlined in Table S4. N: Number of individuals. dEIA, directly immobilized VLP antigens on the enzyme immunoassay plate; sEIA, streptavidin-coated EIA plates with biotinylated VLPs.

**Table S4.** Samples from onco-hematological patients subjected to bone marrow transplantation.

| **Diagnoses** | **Gender** | **Date of birth** | **Serum sampling date** | **Blood or BM sampling date** |
| --- | --- | --- | --- | --- |
| Aplastic anemia | female | 2006 | 27/8/19 | 3/3/15 |
| T-ALL | male | 1985 | 23/8/19 | 8/3/17 |
| AML | male | 1981 | 22/8/19 | 22/8/19 |
| AML | male | 1976 | 19/8/19 | 18/12/18 |
| T lymphoma | male | 1978 | 7/8/19 | 14/2/19 |
| AML | male | 1981 | 5/8/19 | 19/6/19 |
| CML | female | 1978 | 2/9/19 | 9/5/19 |
| MDS | female | 1957 | 21/6/19 | 8/2/19 |
| MDS | male | 1961 | 7/8/19 | 24/7/19 |
| MDS | male | 2009 | 31/7/19 | 31/7/19 |
| AML | female | 1973 | 6/8/19 | 6/8/19 |
| AML | male | 1972 | 7/8/19 | 7/8/19 |
| Hodgkin lymphoma | female | 1979 | 9/8/19 | 9/8/19 |
| B-ALL | female | 1999 | 12/8/19 | 12/8/19 |
| AML | male | 2017 | 13/8/19 | 13/8/19 |
| AML | female | 1957 | 16/8/19 | 16/8/19 |
| Myelofibrosis | female | 1955 | 26/8/19 | 26/8/19 |
| B-ALL | female | 1965 | 26/8/19 | 26/8/19 |
| CML | male | 1956 | 29/8/19 | 28/8/19 |
| AML | male | 1973 | 5/8/19 | 5/8/19 |

*Total DNA was isolated from peripheral blood cells or bone marrow. T-ALL, T cell acute lymphoblastic leukemia; B-ALL, B cell acute lymphoblastic leukemia; AML, acute myeloblastic leukemia; MDS, myelodysplastic syndrome; CML, chronic myeloblastic leukemia; BM, bone marrow.

**Table S5.** Bone marrow samples analyzed by PCR for MVM genomes obtained from onco-hematological patients subjected to allogenic transplantation.

| **Patient #** | **Gender** | **Date** | **Diagnosis** | **Type of sample** |
| --- | --- | --- | --- | --- |
| 1 | Female | 6/8/20 | Ph+ALL | Total BM |
| 1 | Female | 1/14/21 | Follow-up one month post-transplant | Total BM |
| 2 | Female | 2/23/21 | AITL-pre-transplant | Total BM |
| 3 | Male | 9/10/19 | CMML-pre-transplant exam | Total BM |
| 4 | Male | 4/1/19 | AML-Dx | Total BM |
| 4 | Male | 6/11/20 | Relapse AML-four months before transplant | Total BM |
| 5 | Female | 1/23/19 | MDS-Dx | Total BM |
| 5 | Female | 2/10/20 | Follow-up transplanted ten days afterwards | Total BM |
| 6 | Male | 5/19/21 | MDS-five months prior transplant | Total BM |
| 7 | Female | 10/22/20 | T-ALL-Dx | Slide extract |
| 7 | Female | 4/29/21 | T-ALL-pre-transplant | Slide extract |
| 8 | Male | 10/8/20 | AML-Dx | Total BM |
| 8 | Male | 3/8/21 | ALL-pre-transplant | Slide extract |
| 9 | Female | 5/25/20 | AML-Dx | Total BM |
| 9 | Female | 8/31/20 | AML-one month prior transplant | Slide extract |
| 10 | Female | 3/15/21 | AML-relapse | Total BM |

Ph+ ALL, Philadelphia chromosome positive acute lymphoblastic leukemia; ALL, acute lymphoblastic leukemia; AITL, angioimmunoblastic T-cell lymphoma; CMML, chronic myelomonocytic leukemia; AML, acute myeloid leukemia; MDS, myelo dysplastic syndrome; Dx, at diagnosis.

**Table S6.** Human African samples subjected to PCR analysis. Population distribution by demographic parameters

| **Age/**  **Pregnancy** |  | **GHANA** | |  |  |  | | **DRC** | |  |  |  | **TOTAL** | |  |
| --- | --- | --- | --- | --- | --- | --- | --- | --- | --- | --- | --- | --- | --- | --- | --- |
|  | **Female** | | **Male** | **Total** |  | **Female** | **Male** | | **Total** | |  | **Female** | | **Male** | **Total** |
| 0-5 | 9  (32.1) | | 19  (67.9) | 28  (14) |  | 13  (48.1) | 14  (51.8) | | 27  (12.0) | |  | 22  (40) | | 33  (60) | 55  (13.0) |
| 6-13 | 6  (46.1) | | 7  (53.8) | 13  (6.5) |  | 11  (37.9) | 18  (62.1) | | 29  (12.9) | |  | 17  (40.5) | | 25  (59.5) | 42  (9.9) |
| 14-49 | 35  (77.8) | | 10  (22.2) | 45  (22.5) |  | 56  (49.6) | 56  (49.6) | | 113*****  (50.4) | |  | 91  (57.6) | | 66  (41.8) | 158  (37.3) |
| Pregnant | 88  (100) | | 0 | 88  (44) |  | 12  (100) | 0 | | 12  (5.4) | |  | 100  (100) | | 0 | 100  (23.6) |
| >50 | 19  (73.1) | | 7  (26.9) | 26  (13) |  | 16  (37.2) | 25  (58.1) | | 43******  (19.2) | |  | 35  (50.7) | | 32  (46.4) | 69  (16.3) |
| Total | 157  (78.5) | | 43  (21.5) | 200 |  | 108  (48.2) | 113  (50.4) | | 224 | |  | 265  (62.5) | | 156  (36.8) | 424 |

Numbers in parenthesis indicate percentages of individuals. The percentages of female and male have been calculated with respect to the total of each age group. The totals express the percentage of each age group in relation to the country total. *Includes one individual (0.8 %) with unrecorded sex. **Includes two individuals (4.6 %) with unrecorded sex. DRC, Democratic Republic of the Congo.
